# Antegrade anterior column screw placement in the lateral decubitus position utilizing an axial view: a technical trick

**DOI:** 10.1051/sicotj/2020039

**Published:** 2020-11-09

**Authors:** Rahul Vaidya, Ishan Patel, Katelyn Simmons, Kerellos Nasr, Austen Washington

**Affiliations:** 1 Detroit Medical Center Detroit 48201 MI USA; 2 Wayne State University School of Medicine Detroit 48201 MI USA

**Keywords:** Acetabular fracture, Anterior column fracture, Anterior column screws, Kocher-Langenbeck, Posterior approach, Lateral position, Transverse acetabular fracture, Fluoroscopic imaging

## Abstract

The placement of anterior column screws is a useful procedure and has standard views when placing this screw in the supine position. Feng et al. described an acetabular anterior column axial view for patients in the supine position for a placement of a retrograde anterior column screw [J Orthop Surg (Hong Kong) 25, 2309499016685012]. However, many acetabular fracture surgeries are performed in the lateral decubitus position due to a variety of reasons. Placing an antegrade anterior column screw in this position is difficult due to an unfamiliarity of the optimal fluoroscopic images. The purpose of this article is to describe a novel technique to obtain appropriate imaging to safely place an anterior column screw while the patient is in the lateral decubitus position.

## Introduction

The ability to place a percutaneous anterior column screw is a powerful and an effective tool in the armamentarium of an orthopedic traumatologist. Descriptions of this technique are well described with standard intra-operative fluoroscopy views, and the patient in the supine position [1–7]. Traditionally, these cannulated screws are placed by alternating between an obturator–outlet view and an iliac-inlet view to safely pass the guide wire within an osseous corridor [1–4]. Alternative views have been proposed by Cunningham et al. to minimize discomfort and increase the reliability with this procedure [8]. Other authors have described using either an intraoperative CT or a CT-based navigational approach to facilitate this surgery [9, 10]. Identifying an appropriate starting point for the guide wire comprises one of the main difficulties with this procedure [8]. Feng et al. recently described an axial view projection of the anterior column based on computer modeling for placing this screw in a retrograde fashion with the patient positioned supine [11–13]. While most previous work has described performing this procedure using the standard fluoroscopy views, this is almost always done with the patient in the supine position and either antegrade or retrograde [1–8].

Acetabular fracture patterns with a transverse component are often fixed with the patient in the lateral decubitus position using a posterior approach (posterior wall component, fracture displacement, etc.). In this position, it is difficult to obtain a pelvic inlet view due to the position of the pelvis, obstruction from the operating table arm board, positioning devices, or the patient’s abdomen [5].

The purpose of this paper is to describe a simple technique to reproduce the axial view of the anterior column in the lateral decubitus position to facilitate the placement of anterior column screws using fluoroscopy.

## Methods

### Sawbones

**Figure 1:**
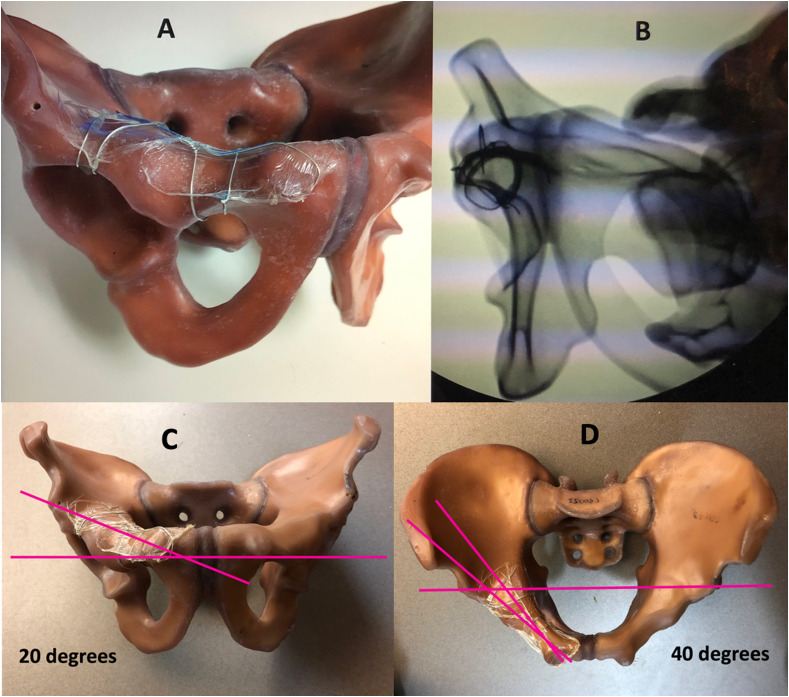
(A) Pelvic saw bone model with wire encircling the pubic ramus, pelvic brim, the dome of the acetabulum and the medial border of quadrilateral plate. (B) A fluoroscopic view of this model with the axial view for the anterior column screw. (C) The superior ramus is inclined 20° off the horizontal in the AP plane. (D) The superior ramus is inclined 40° off the horizontal plane.

Similar to the technique described by Judet and Letournel (Acetabular Tome), we first aimed to radiographically define and describe an axial view of the anterior column. Using a pelvic Sawbones model (Sawbones USA, Vashon Island, Washington), we placed thin wires around the superior pubic ramus in three critical positions: (1) the pubic root, (2) the most minimal diameter of the ramus, and (3) the distal portion of the ramus. Subsequently, wires were also placed along the pelvic brim, the dome of the acetabulum, and the medial border of quadrilateral plate (Figure 1A). The Sawbones pelvis was then secured on a radiolucent surgical table (Mizohi OSI, Union City, CA, USA) in the lateral decubitus position and the fluoroscopic C-arm image intensifier (General Electric OEC 9900) was positioned in a standard neutral upright fashion perpendicular to the surgical table. The anterior axial column projection was recreated in this position and the angles necessary to reproduce it were measured (Figure 1B). To account for the inclination of the superior rami in the outlet view, the image intensifier was moved 20° cranial (Figure 1C, this movement is similar to performing an inlet view when the patient is in the supine position). Lastly, the image intensifier was then rotated 40° over the pelvis to account for rami orientation in the inlet view (Figure 1D). We then fine-tuned the image with minor movements of the image intensifier to obtain a “perfect circle” of the wires delineating the axial view of the anterior column (Figure 1B).

**Figure 2:**
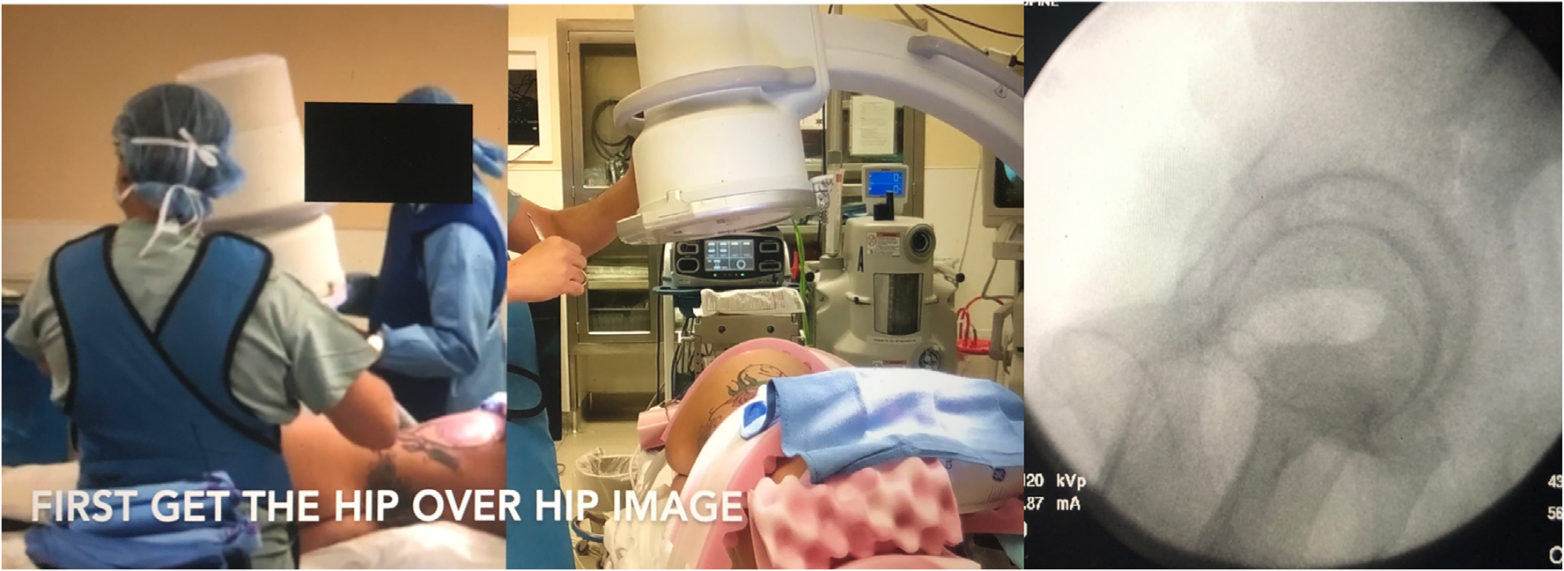
The patient is in the lateral decubitus position, the c-arm is positioned to get the hip over hip image which is the starting point. The fluoroscopic image shows the two hips overlying each other.

**Figure 3:**
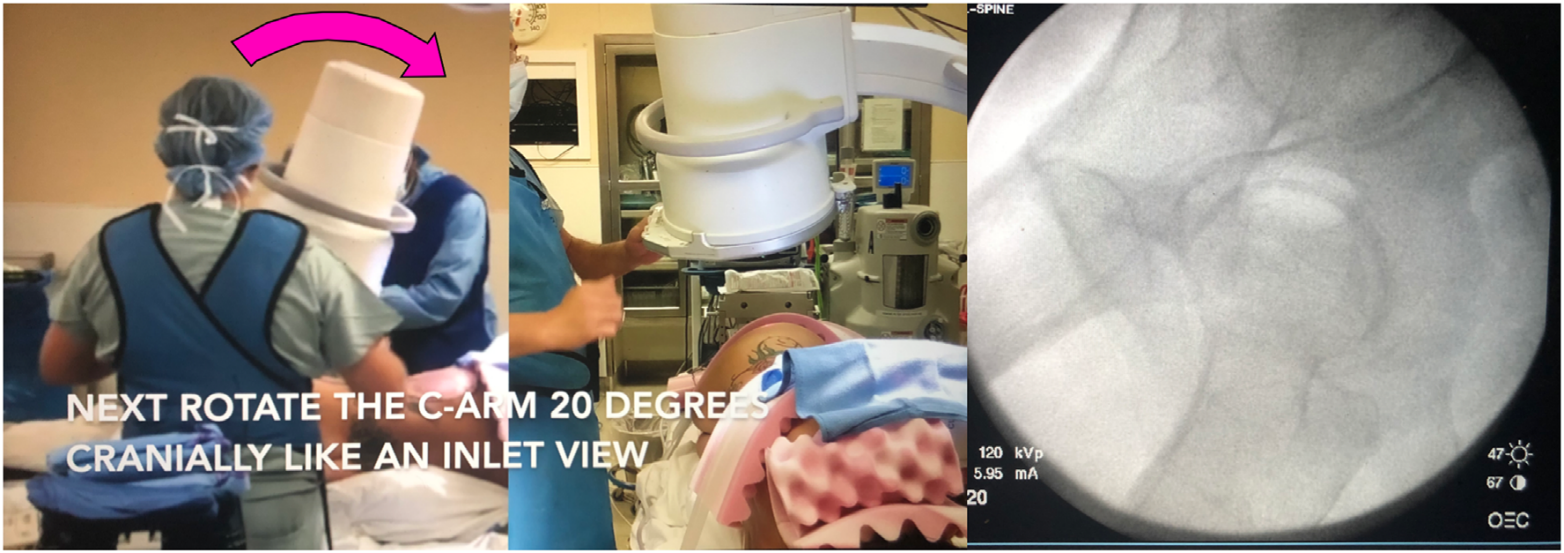
Step 2: rotate the c-arm 20° clockwise. The fluoroscopic image obtained.

Attempts were made to reproduce the same view in patients placed in the lateral decubitus position who were undergoing acetabular surgery. This was found to be very difficult and time consuming. The only image that was easy to reproduce in the lateral position was the 2 hips overlying each other. This was independent of the tilt of the pelvis forward, backward, or the pelvis’ natural tendency to angle in the coronal plane. This became the starting image (Figure 2). The c-arm is then rotated 20° clockwise around this plane (Figure 3) for the left hip and counter clockwise for the right hip. The c-arm is then rotated back 40° over the top in the opposite plane (Figure 5). The axial view of the anterior column comes into plane view and then it is like locking a nail distally with “perfect circles.”

**Figure 4:**
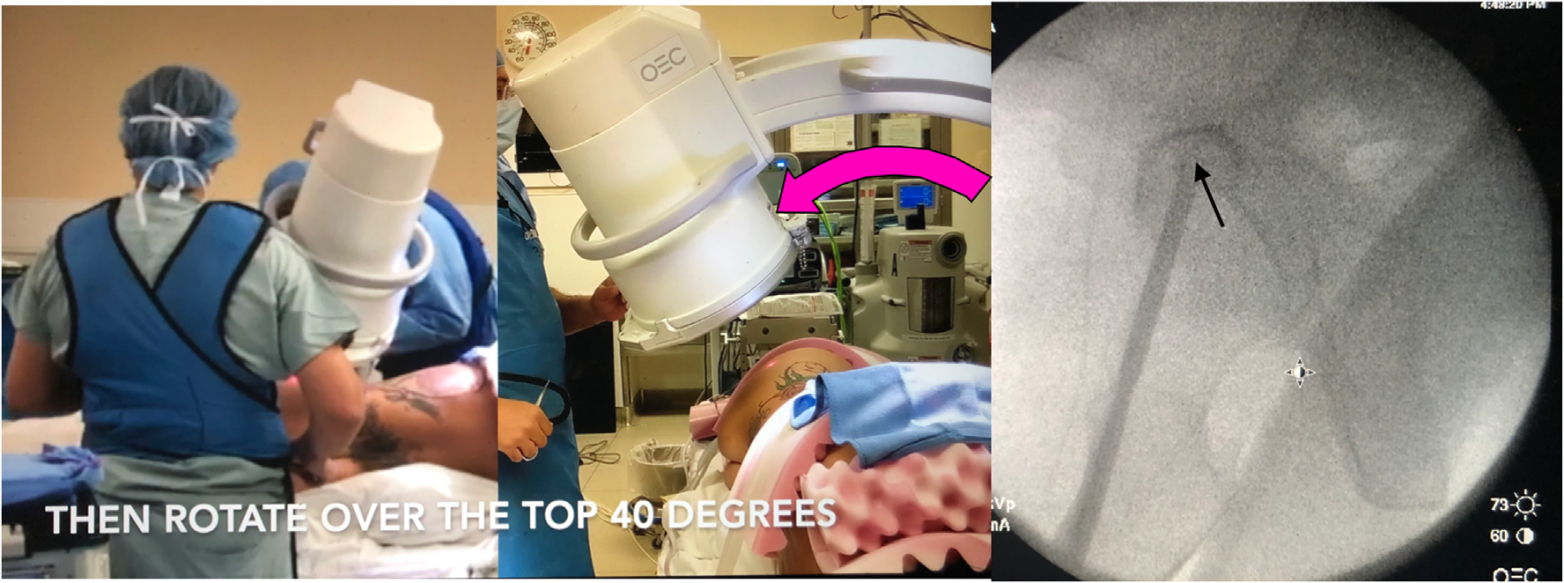
Step 3: the c-arm is rotated 40° over the top. Fluoroscopic image showing the axial view of the acetabular anterior column

**Figure 5:**
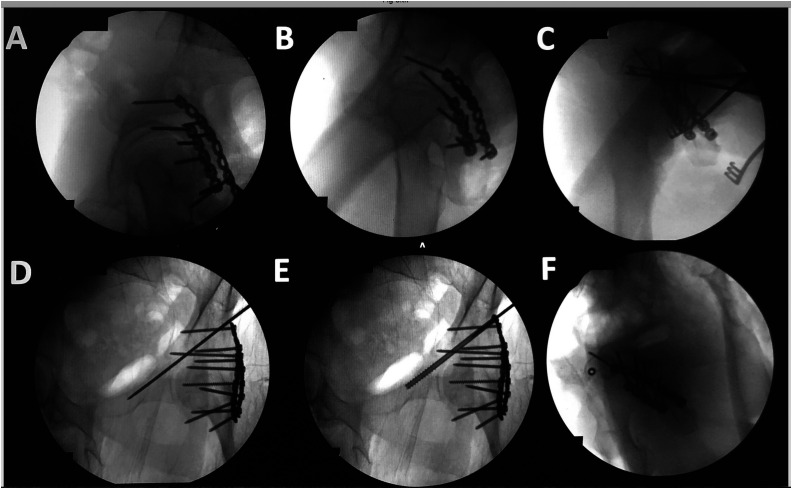
Placing the antegrade anterior column screw. (A) Hip over hip image. (B) Clockwise rotation of the c-arm 20° fluoro shot. (C) The c-arm is rotated over the top 40° with the axial view of the anterior column shown with a 2.8 mm k-wire in its center. (D) K wire drilled down the anterior column on the obturator view. (E) Placement of a 6.5 mm canulated screw. (F) The axial anterior column view to check the placement of the screw outside the acetabulum.

The starting point for this screw is in the middle of this fluoroscopic shape (Figure 4 arrow). The direction of the wire should be in line with this view. A jamshidi needle or a vertebroplasty cannula can be used as it is shorter than the k-wire for 6.5 or 7.3 cannulated screws and can be malleted through the cortex and then can be tapped through the anterior column. We then place a 2.8-mm k-wire with the blunt side in and tap it across the fracture, which may bounce off the cortical bone as described by many previous authors (Figure 5D) [1–6, 8]. The inlet view can be difficult unless you have a radiolucent table that can tilt to the right or left as the pelvis often falls forward in the lateral position. The obturator outlet view is easier to obtain and we use this view to advance the wire and screw down the ramus (Figure 5E). Reduction of the fracture is required for this procedure and can be done with a mini open procedure. Alternatively, a wire can be passed across the gap and then the fracture reduced by a partially threaded cannulated screw [1–6, 8]. This procedure was performed on 16 consecutive operative cases where we felt this procedure would be useful. A CT scan was performed post surgery to confirm the proper position.

## Results

Sixteen consecutive cases were included. Their demographics are presented in Table 1. These patients were all Transverse or T-Type fractures where the major displacement was of the posterior column. Several of them also incurred a posterior wall fracture and some of them a concomitant pelvic ring injury (Table 1). In all cases, a Kocher Langenbeck approach was utilized for the treatment of the acetabular fracture with the patient in the lateral decubitus position. The fracture was reduced first and subsequently stabilized with an appropriate length pelvic reconstruction plate (Synthes, Paoli, PA) and cortical screws along the posterior column. The anterior column portion of the fracture was then addressed with any required reduction and then fixated with an antegrade anterior column screw via the technique described above. A CT scan was performed on all of the 16 patients to assess the technique, which showed the screw remained inside the osseous corridor and did not penetrate the joint surface. Representative cases are presented in the Supplementary Material (Supplement 1).

**Table 1:** Sixteen consecutive cases.

Patient	Gender	Age	BMI	OTA class	Length of surgery/anterior column time (min)
1	M	28	31.6	62B1.2b + 61B2.3	333/21
2	M	23	23.6	62B1.2c + 61b3.3	549/24
3	F	61	24.9	62B1.2c	354/24
4	M	34	45.2	62B2.2c	255/21
5	M	59	30.9	62B2.2 + 61B3.1b	260/24
6	M	26	19	62B2.2b + 61C1.3	373/31
7	M	25	21.6	62B1.2 + 61B3.1b	706/33
8	M	55	20.3	62B2.2 + 61B3.1b	191/32
9	M	59	20.6	62B1.2 + 61B3.1b	324/21
10	F	55	39.1	62B1.2	120/17
11	M	44	25.9	62B1.2c	443/23
12	M	47	26	62B1.2b	343/38
13	M	21	29.3	62B1.2b	174/32
14	F	57	21.3	62B1.2b	244/26
15	M	69	28	62B1.2b	176/19
16	F	47	25.4	61B2.1 + 62B1.2c	265/36

## Discussion

In this series, we first devised and then demonstrated a safe and effective fluoroscopic technique to percutaneously place an anterior column screw for the treatment of acetabular fractures while the patient is in a lateral decubitus position. We utilized an axial view of the anterior column to not only provide an accurate starting point for the guide wire, but also to precisely guide its trajectory. We then subsequently confirmed placement of the screw on standard iliac-inlet and obturator-outlet views in 16 patients with appropriate acetabular fractures. In some cases the obturator outlet view and the axial anterior column view are the only perspectives available.

Patients undergoing surgery for an acetabular fracture are positioned either supine, lateral decubitus or even prone, depending on the fracture pattern and technique of the treating surgeon. Previous works [1–11] have described the placement of anterior column screws with the patient either in the supine or prone position due to the relative ease of fluoroscopic imaging. Most orthopedic trauma surgeons are comfortable with treating transverse or posterior column and wall fractures through a posterior approach (Kocher Langenbeck or Gibson) necessitating the lateral decubitus position. Subsequently many struggle with easily obtaining, in a reproducible manner, the fluoroscopic images required to safely place anterior column screws in an antegrade fashion.

We feel that this technique is generalizable to the majority of patients with anterior column acetabular fracture components that are treated in the lateral position. While the treating surgeon must always exercise caution and judgement if he or she is not certain of osseous markers and a safe screw passage, we think that this methodology gives a good framework for identifying the appropriate structures in the majority of cases. This study is limited in that this only demonstrated on 16 patients; however, there was a distribution of fracture morphology and patient body habitus. Also, we were successful in utilizing this technique in all of the consecutive patients, and we did not encounter any patients, or subsequent anatomy, that would preclude this method.

## Conclusion

In conclusion, this technique of an axial anterior column view is an indispensable tool to utilize in placing antegrade anterior column screws for acetabular fractures when the patient is in the lateral decubitus position. This stepwise methodology is a powerful addition to the armamentarium of the surgeon treating these fractures and reduces the frustration with obtaining the correct imaging.

## Supplemental Material

*Supplement 1*. Supplemental digital content 1. Supplementary material is available at https://www.sicotj-journal.org/10.1051/sicotj/2020039/olm.

## Conflicts of interest

The authors declare that they have no conflicts of interest in relation to this article.
