# Supplementary material for: Antegrade anterior column screw placement in the lateral decubitus position utilizing an axial view: a technical trick
Source: SICOT J. 2020 Nov 9;6:43. doi: 10.1051/sicotj/2020039 (PMC7735812; doi:10.1051/sicotj/2020039)

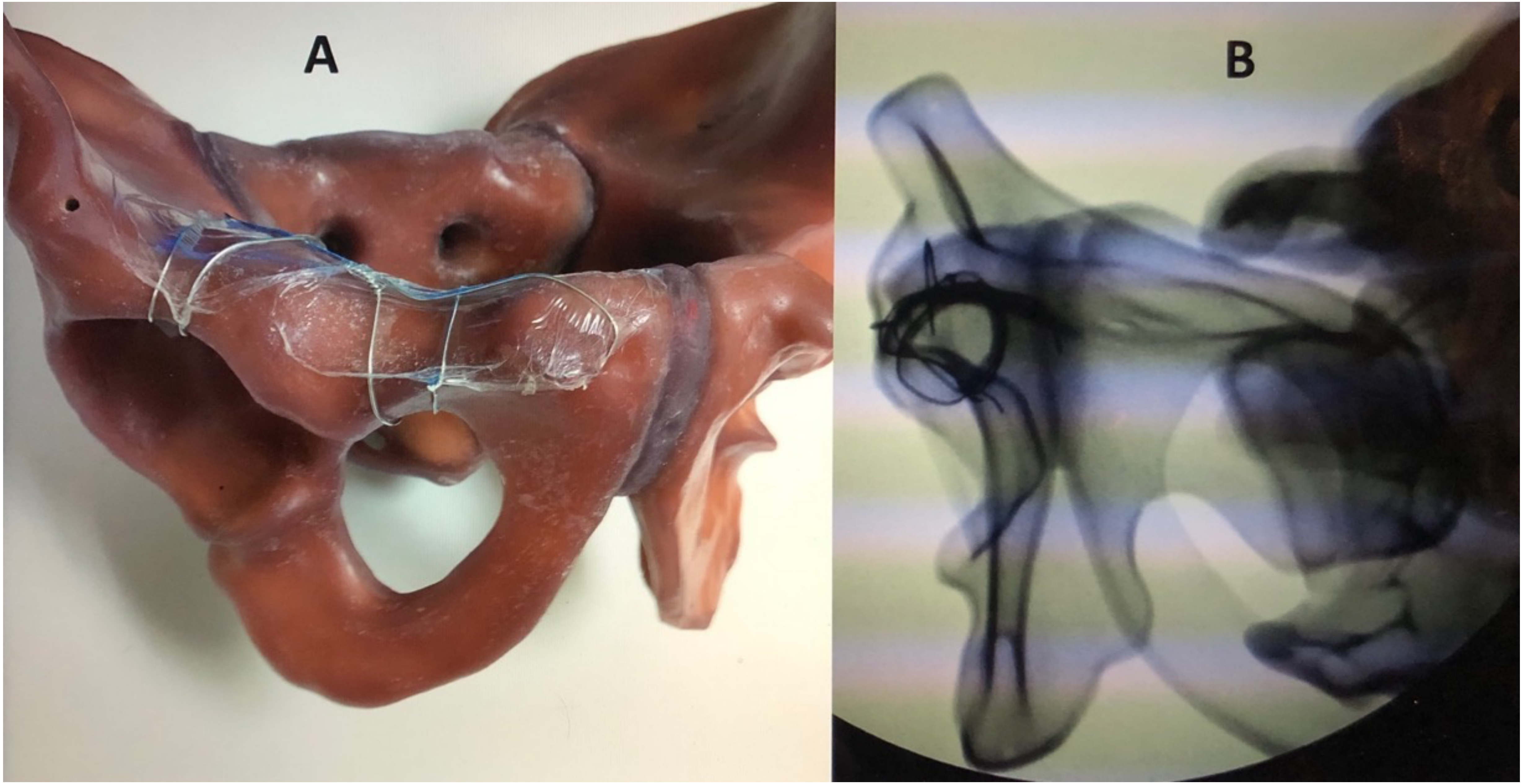
A
B

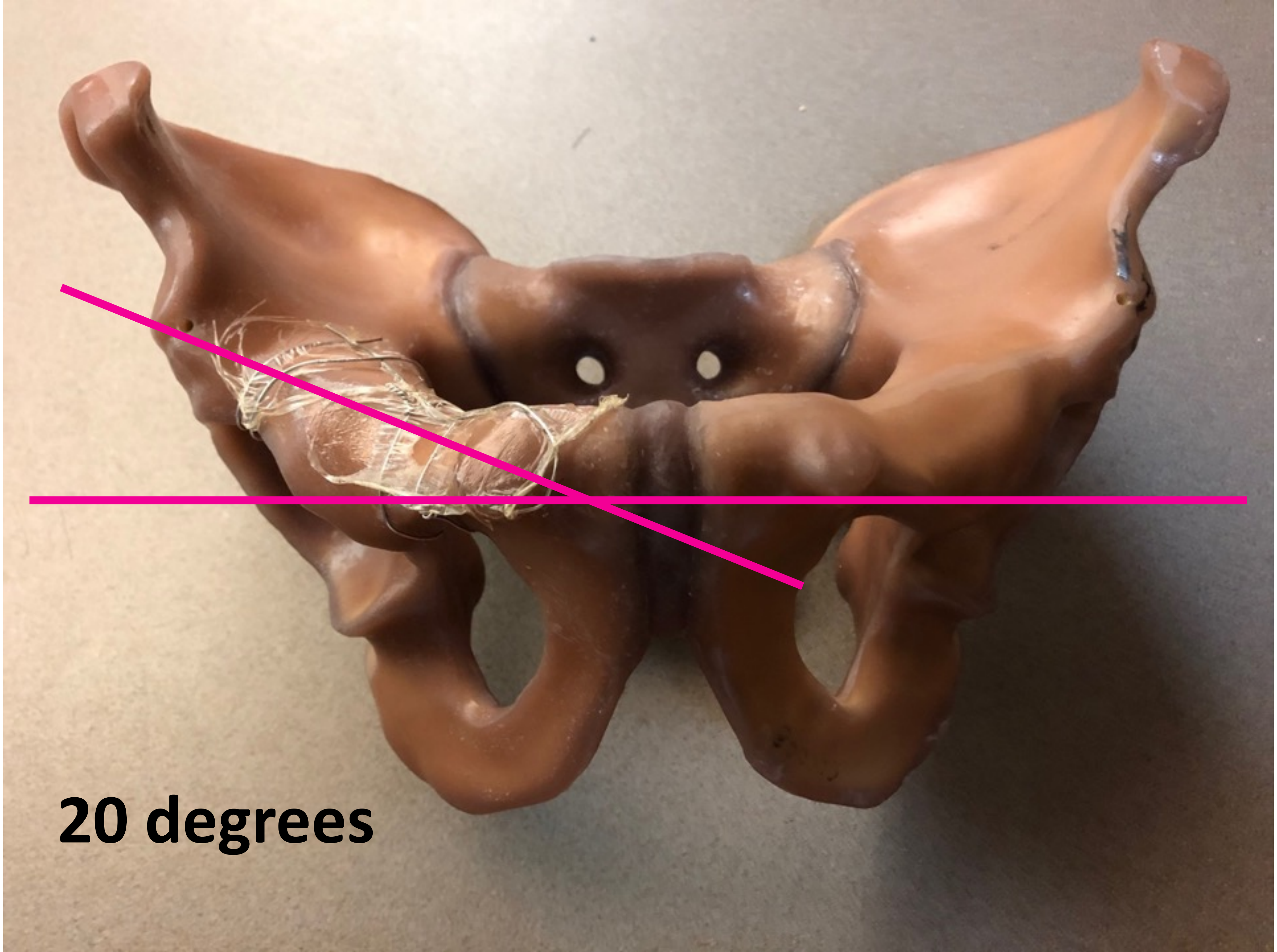
20 degrees

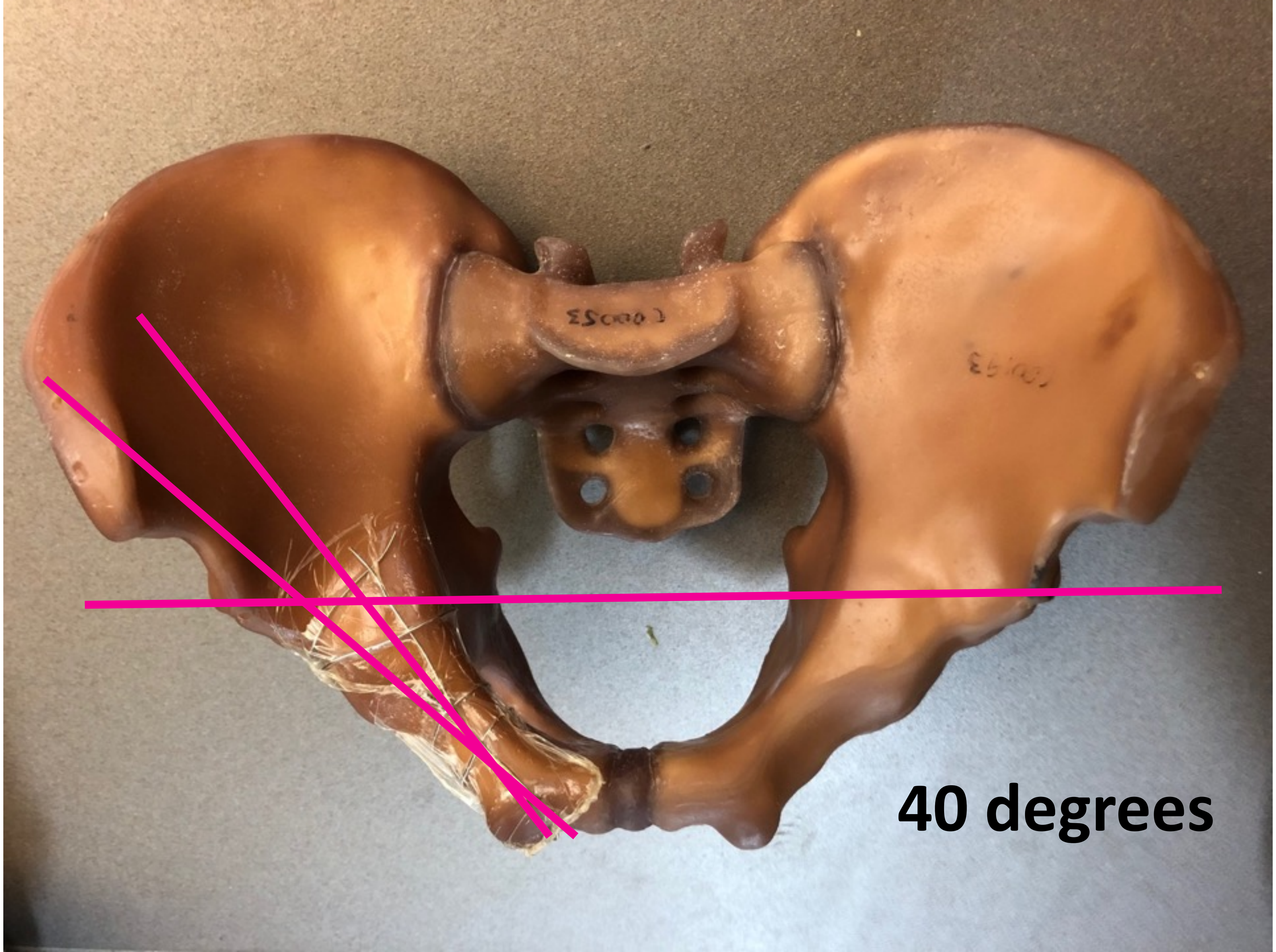
40 degrees

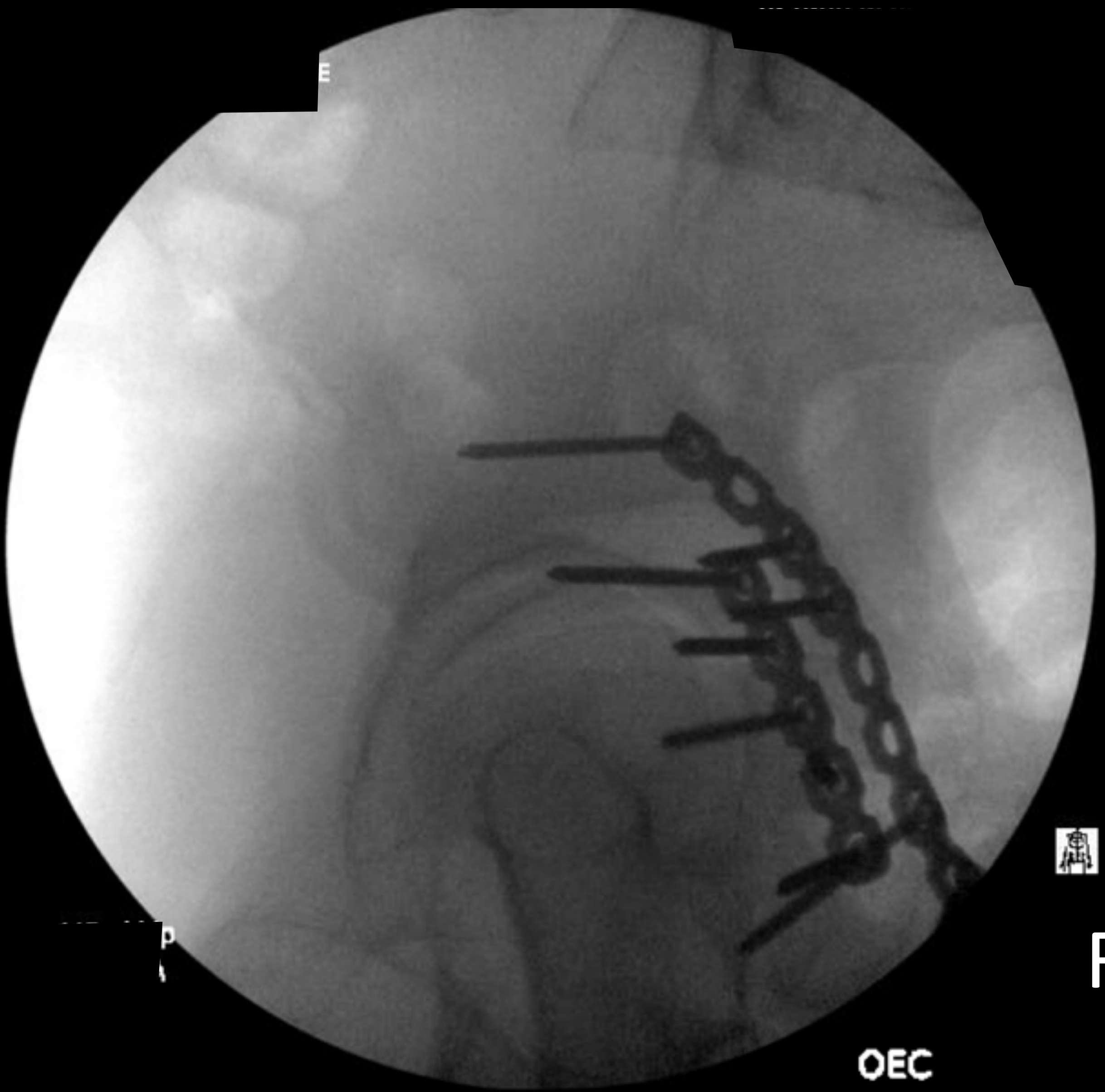

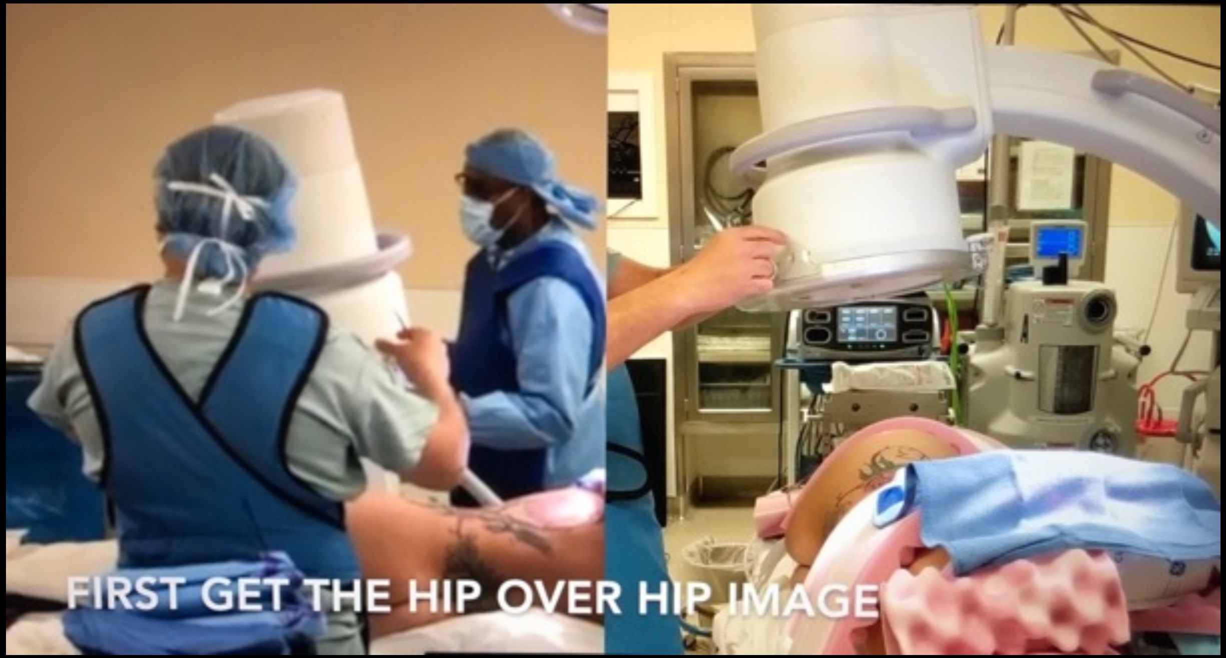

First get the hip over hip image

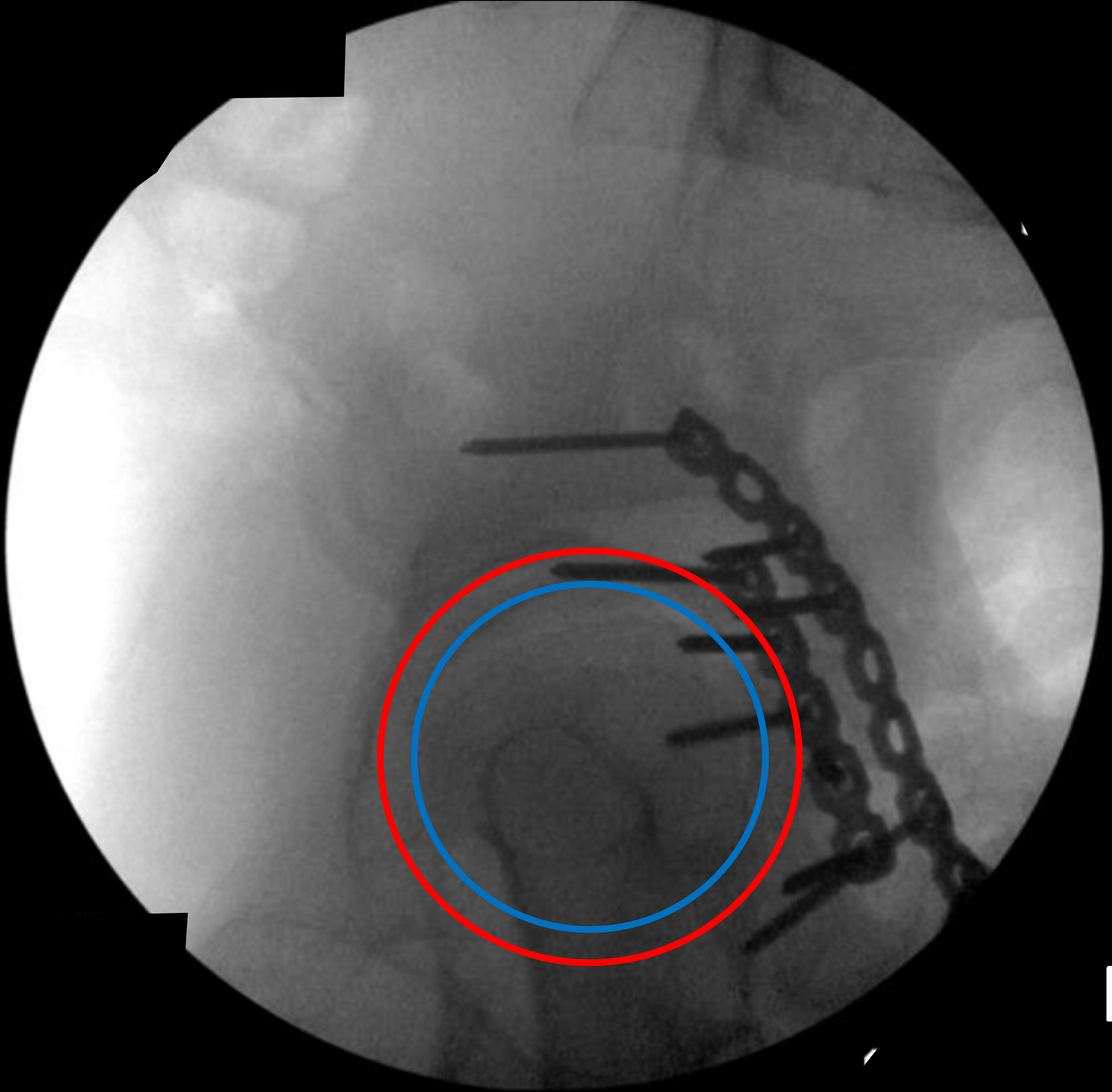

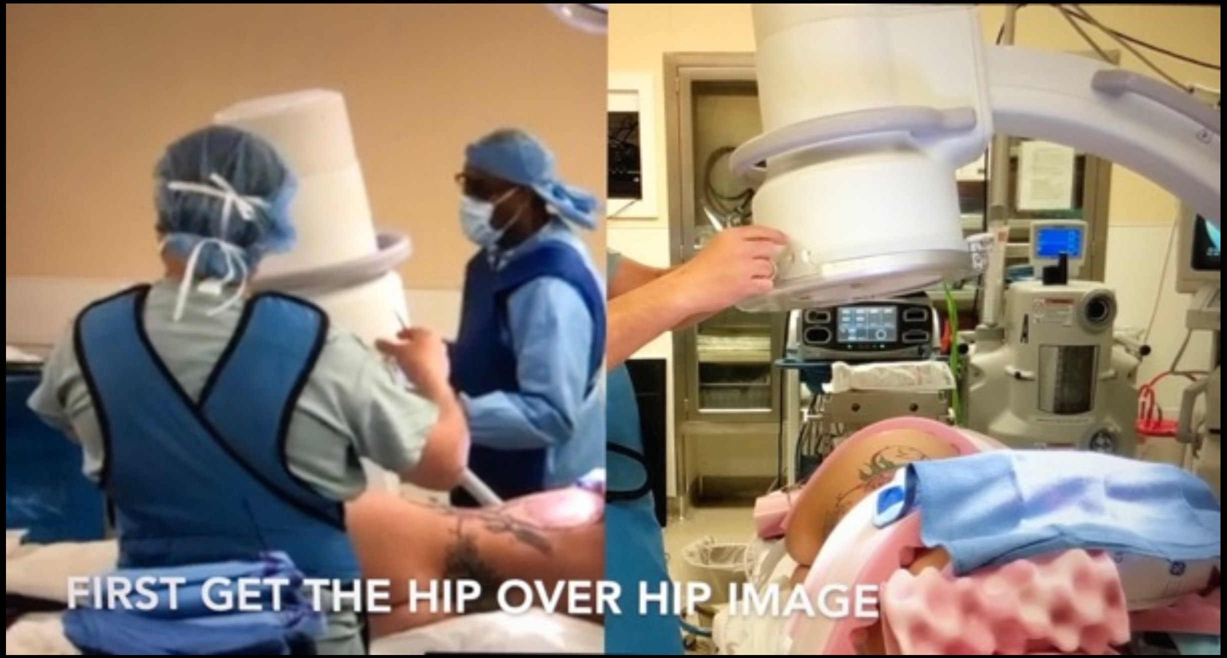

First get the hip over hip image

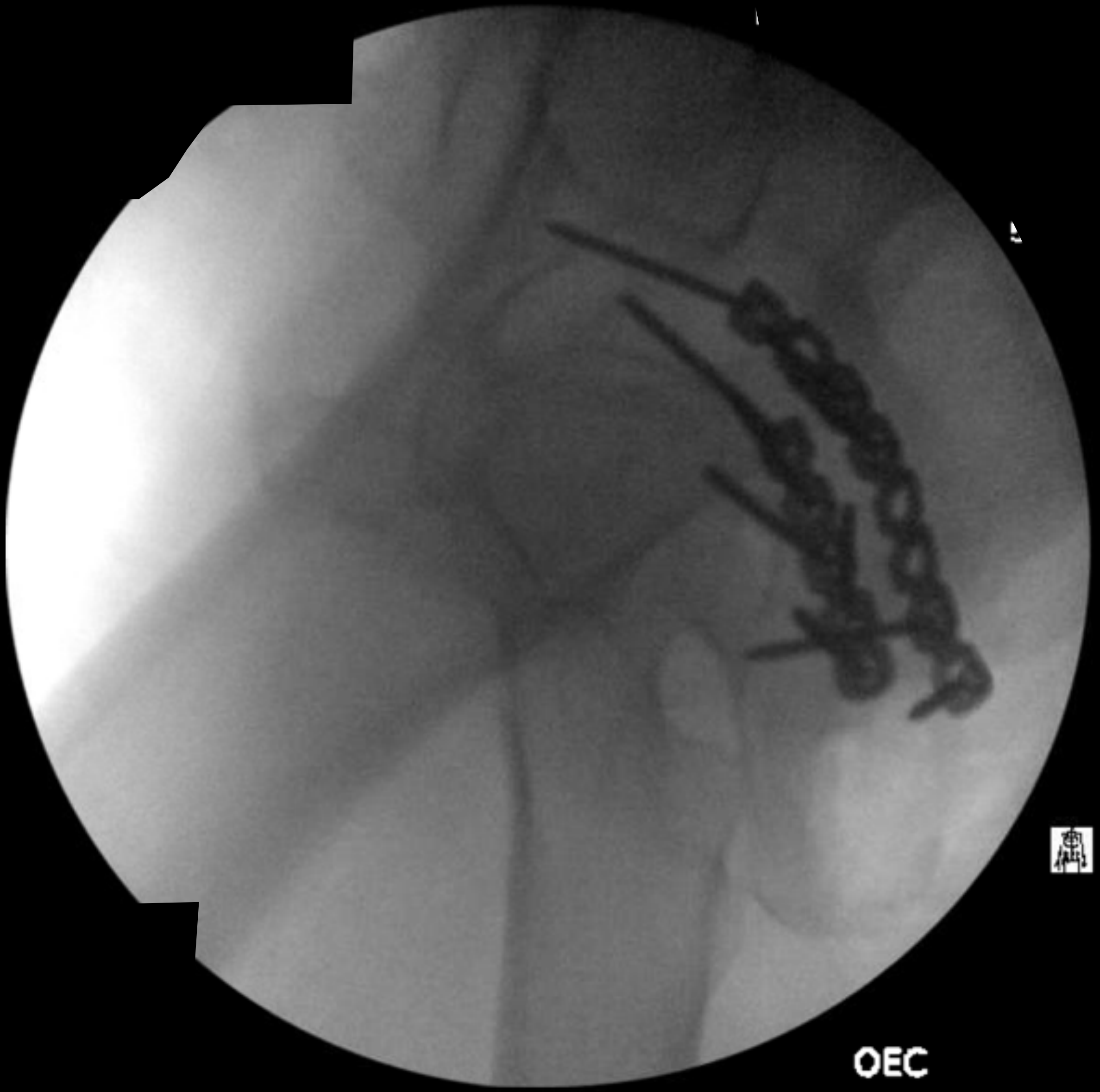

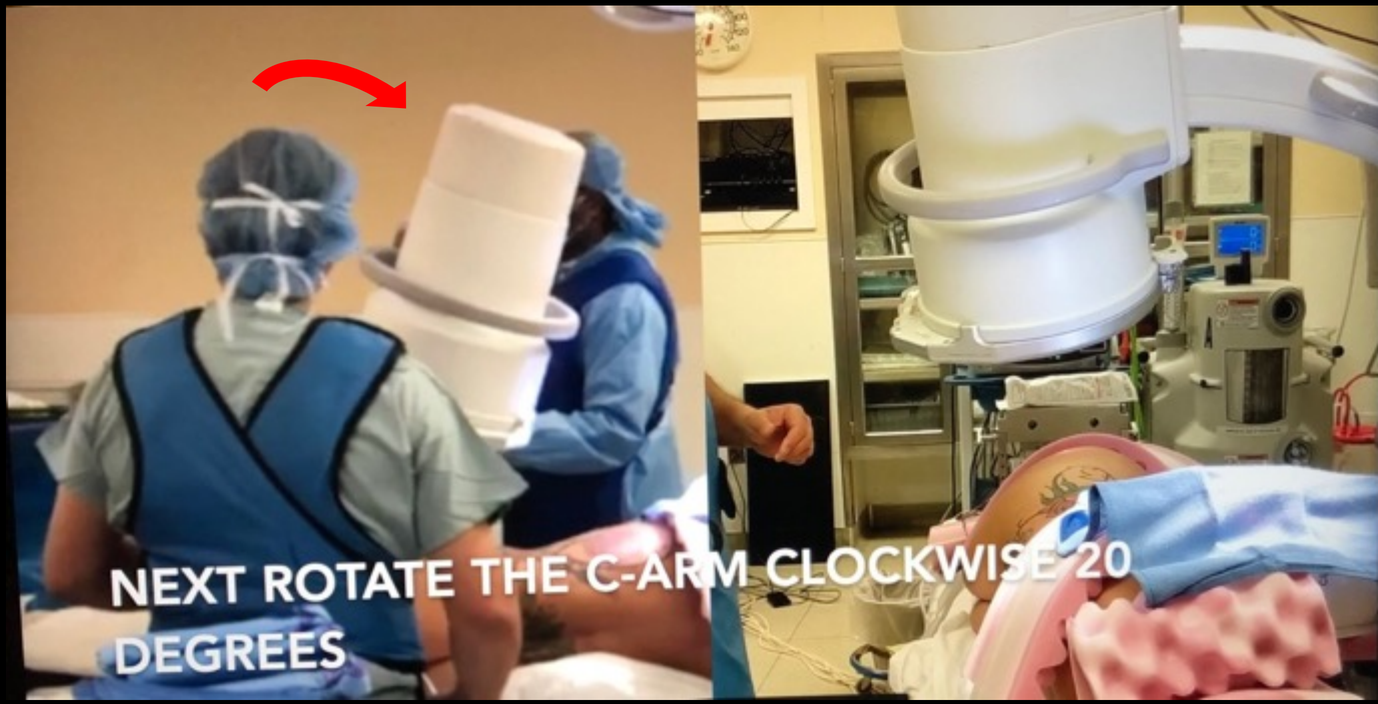

Rotate the c-arm 20 degrees clockwise for the left hi
and counterclockwise for the right hip

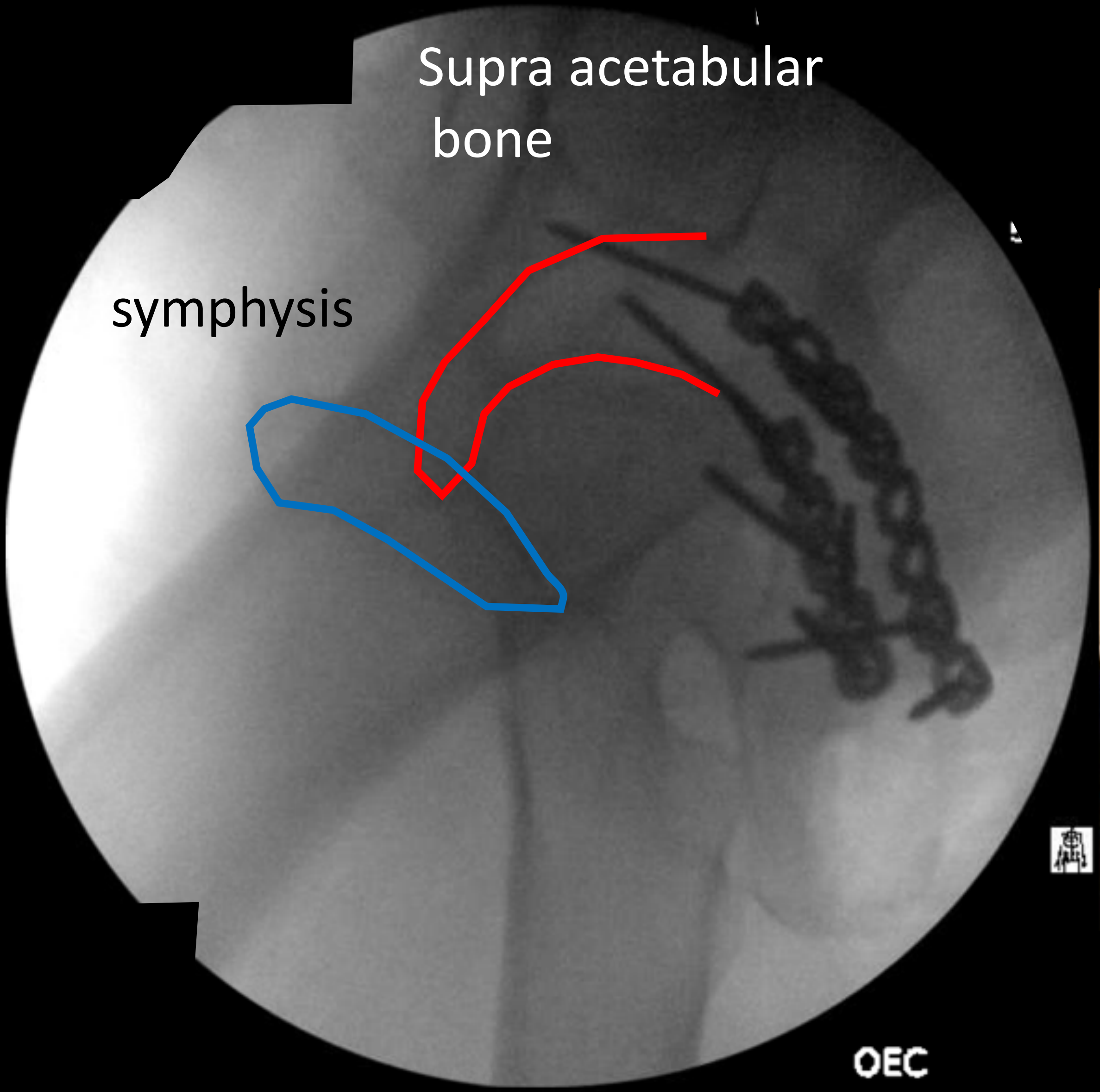

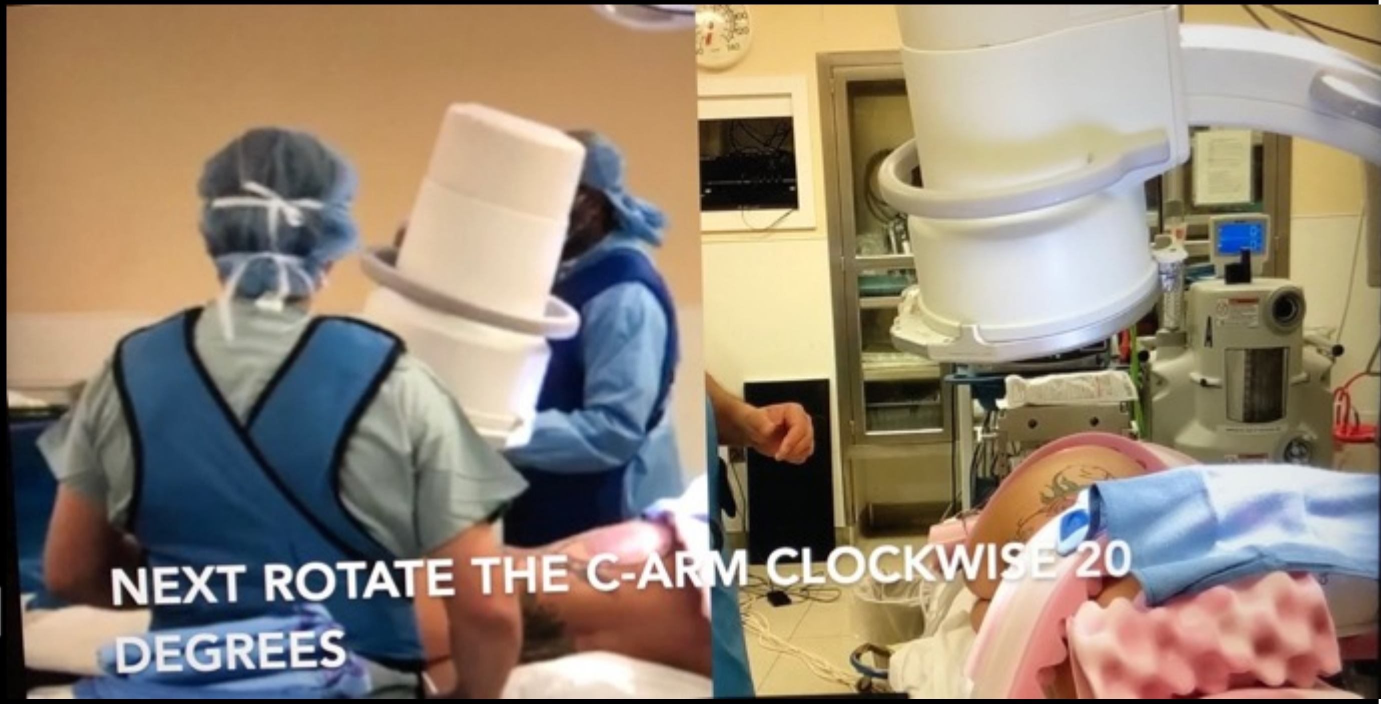

You can see the x-ray shadows of the supraacetabular bone and the symphysis

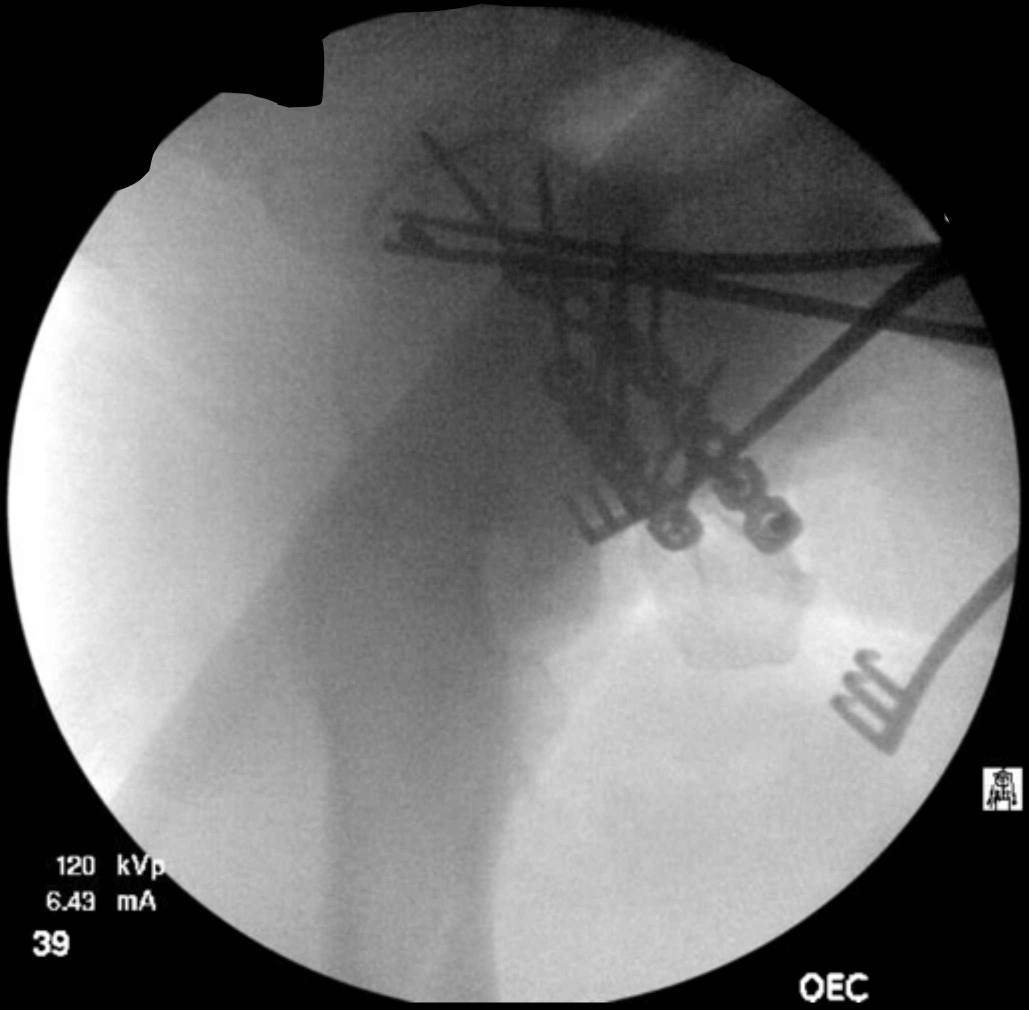

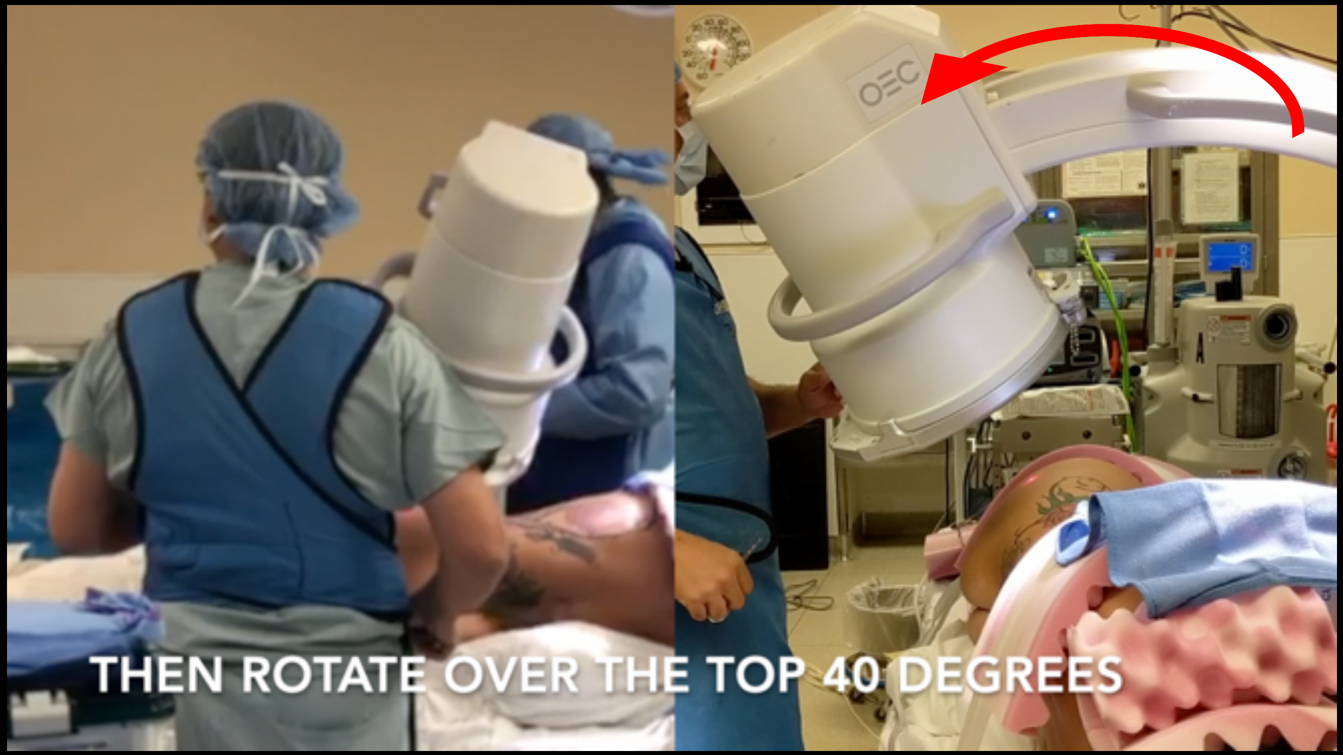

Rotate the c-arm over the top towards you about 40 degrees

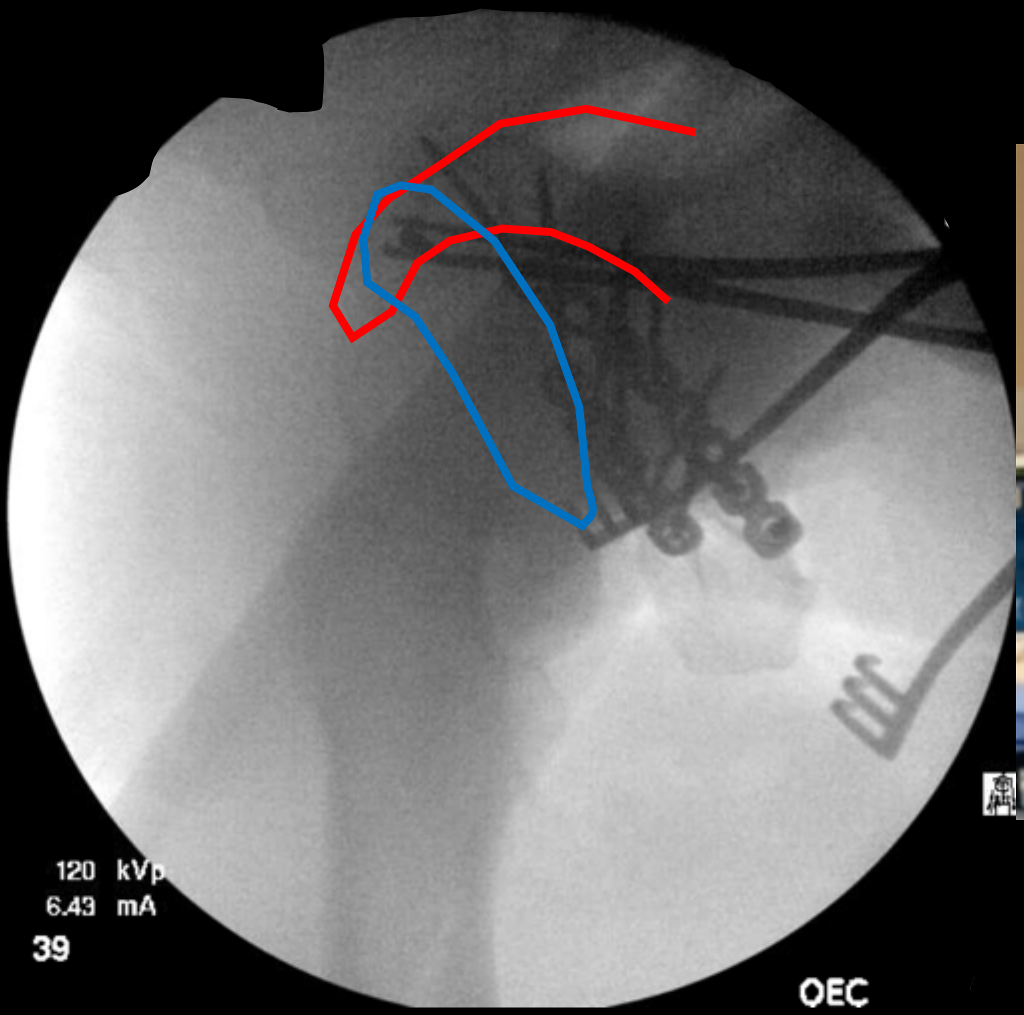

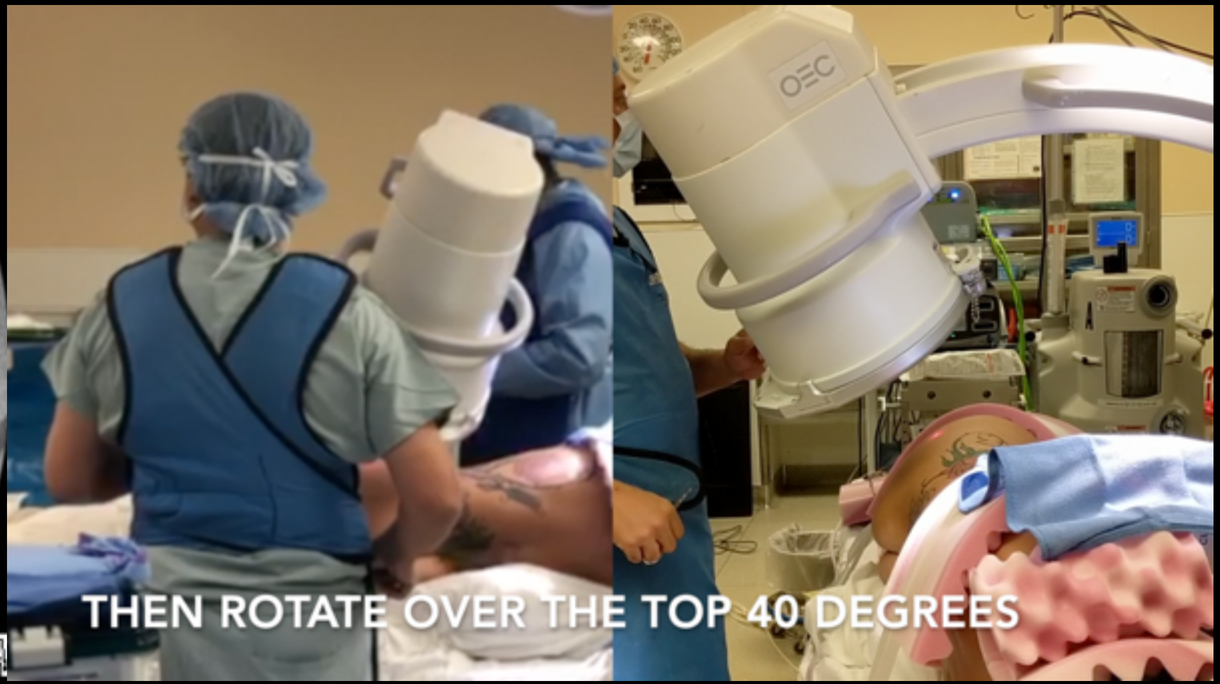

You can see the overlap of the supraacetabular bone shadow with the symphysis

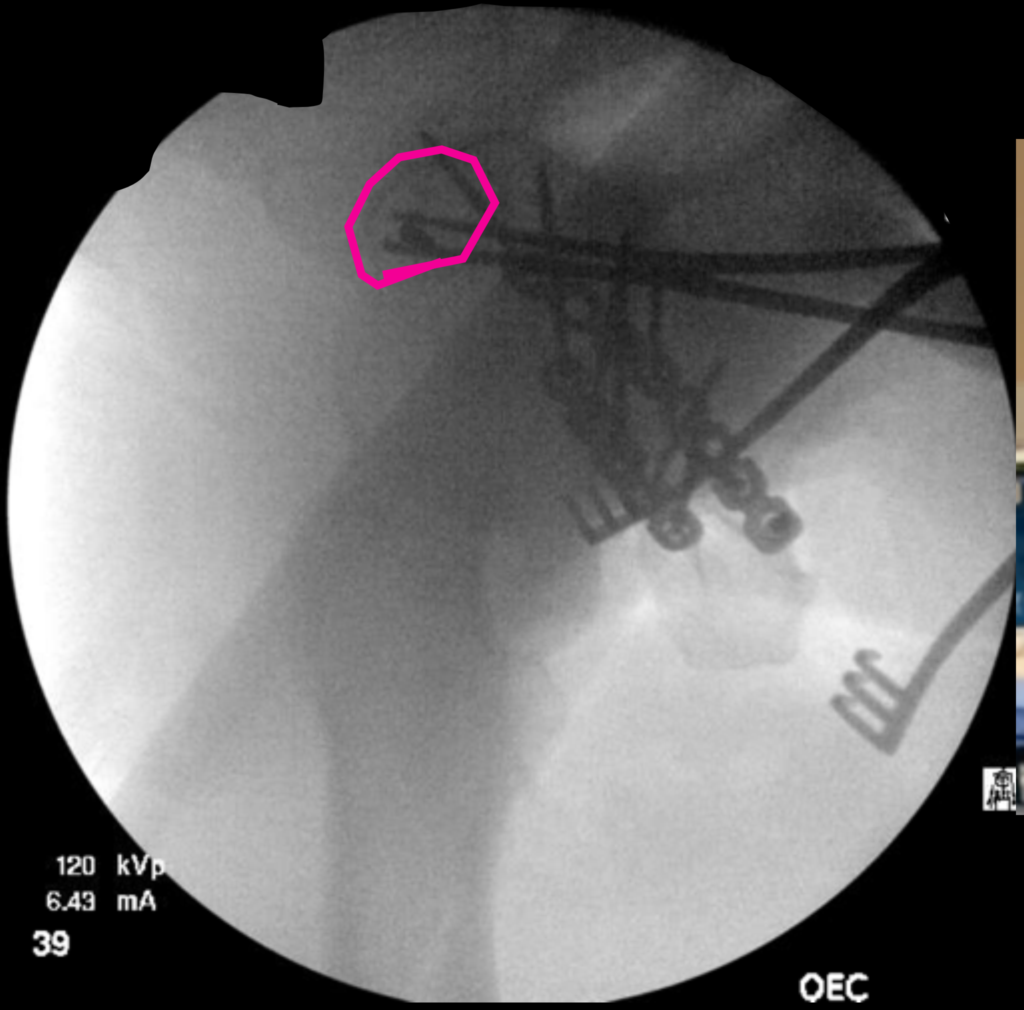

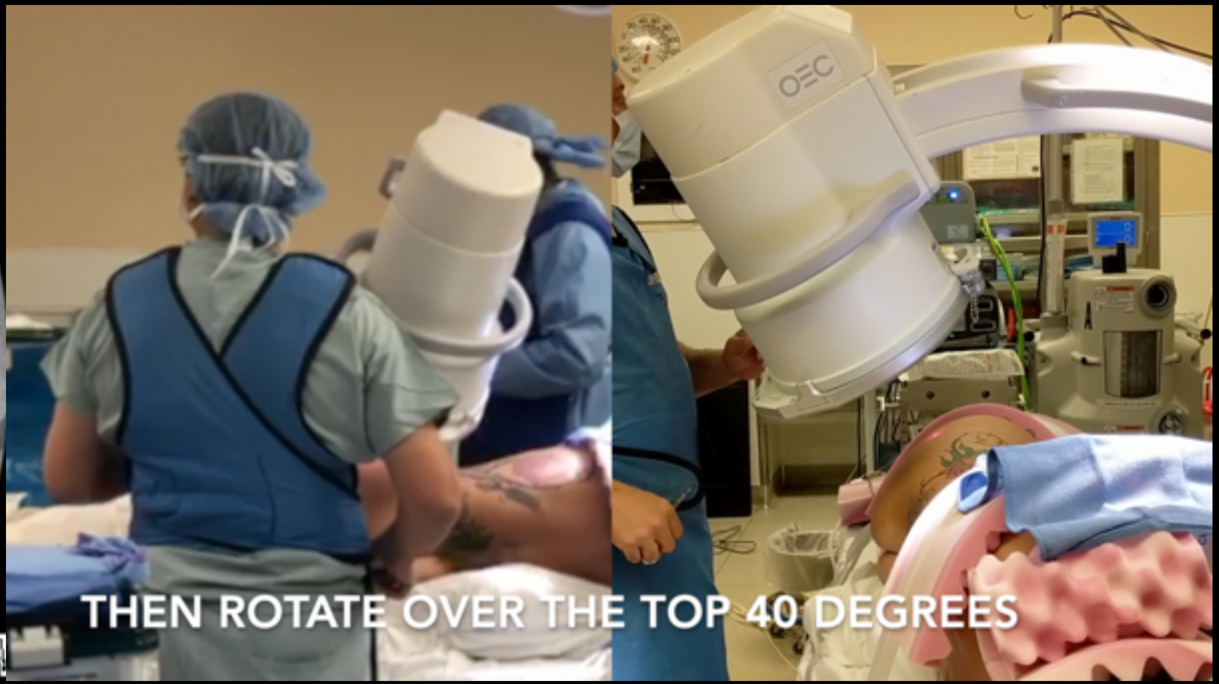

The overlapped area is the safe area to start at and it is like perfect circles when you are locking a nail

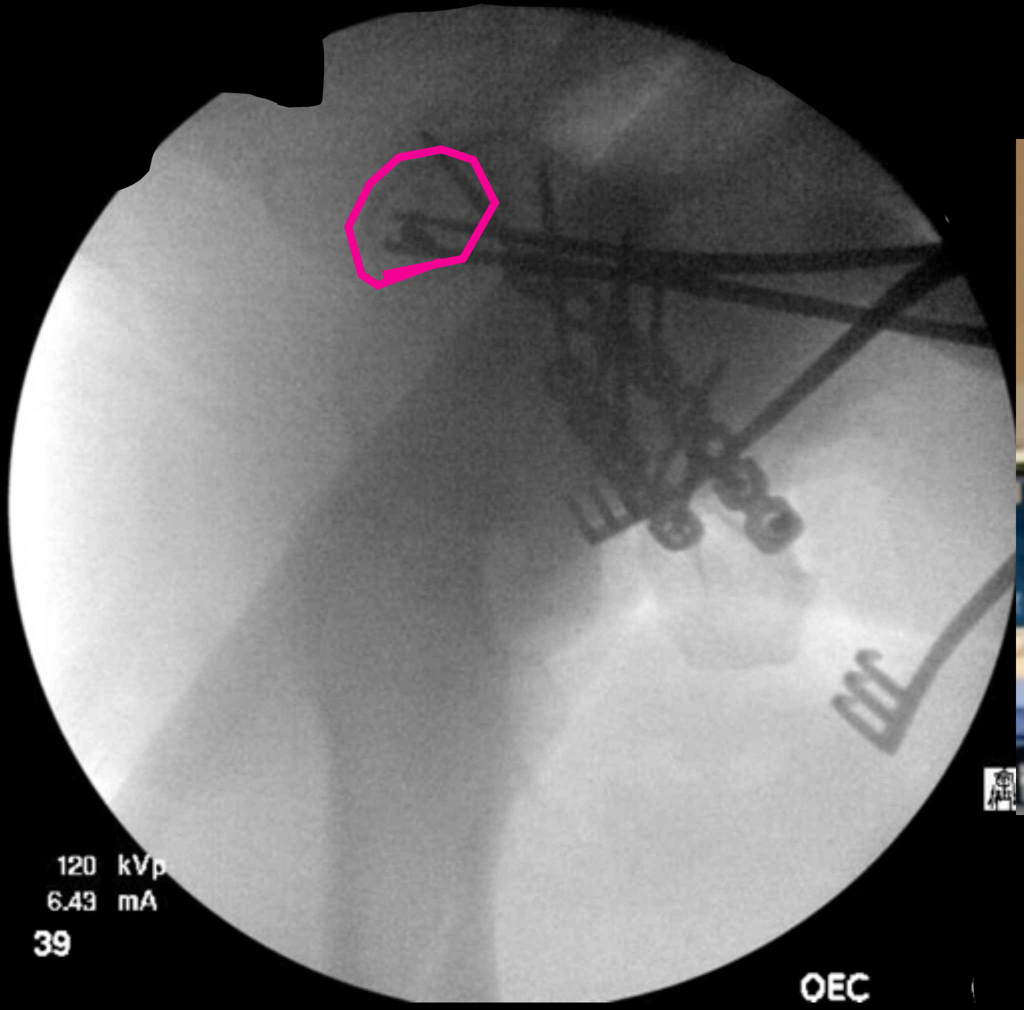

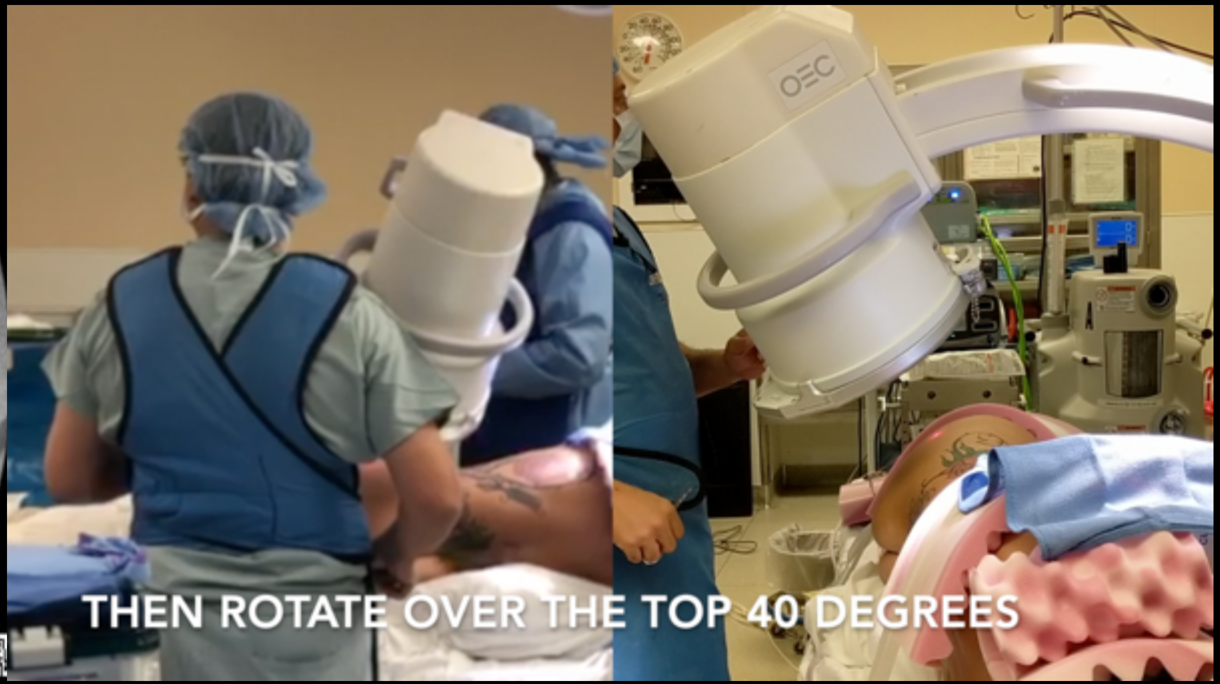

Insert the wire staying in the middle of the shadow

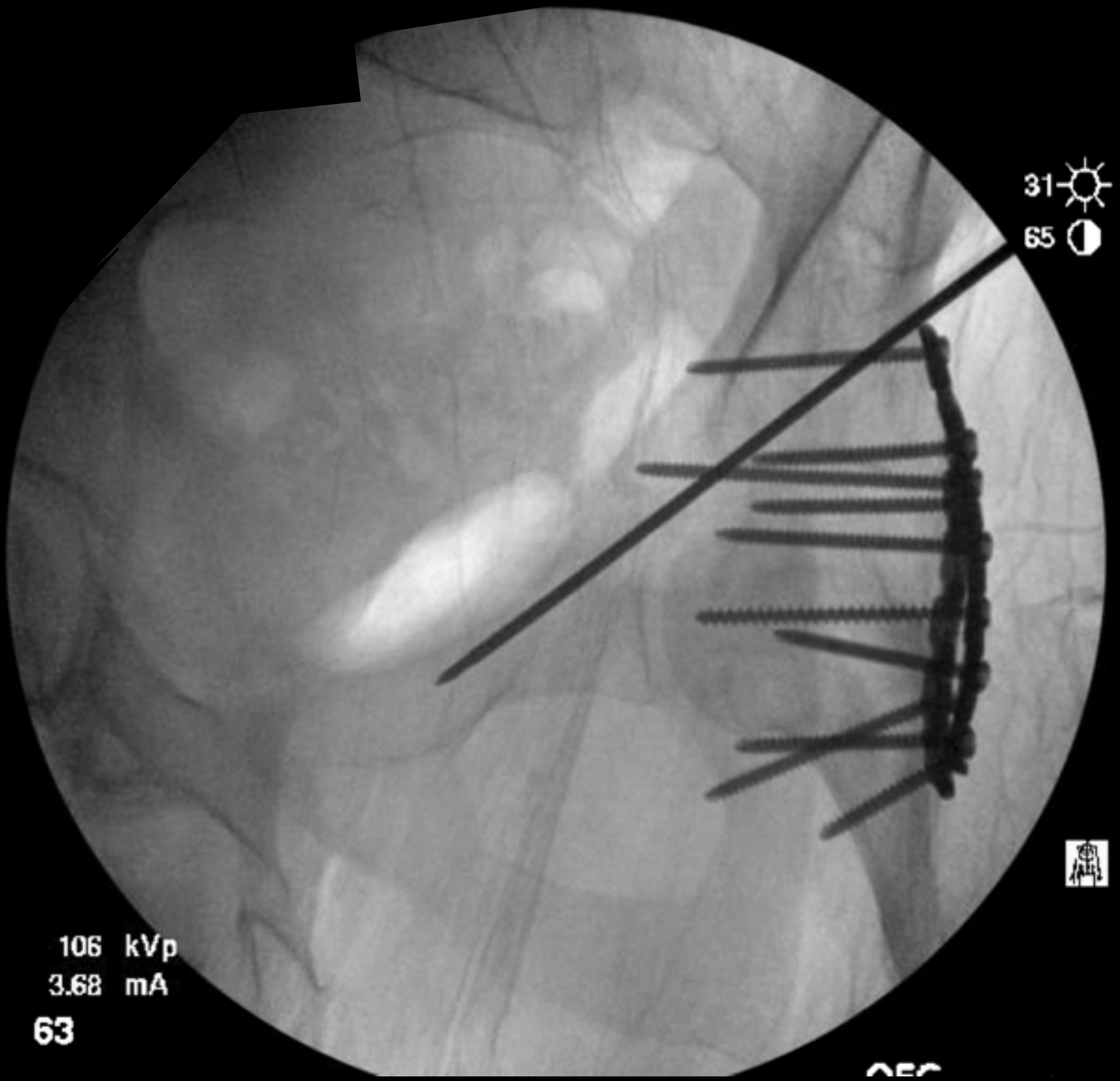

Check position with the obturator outlet view

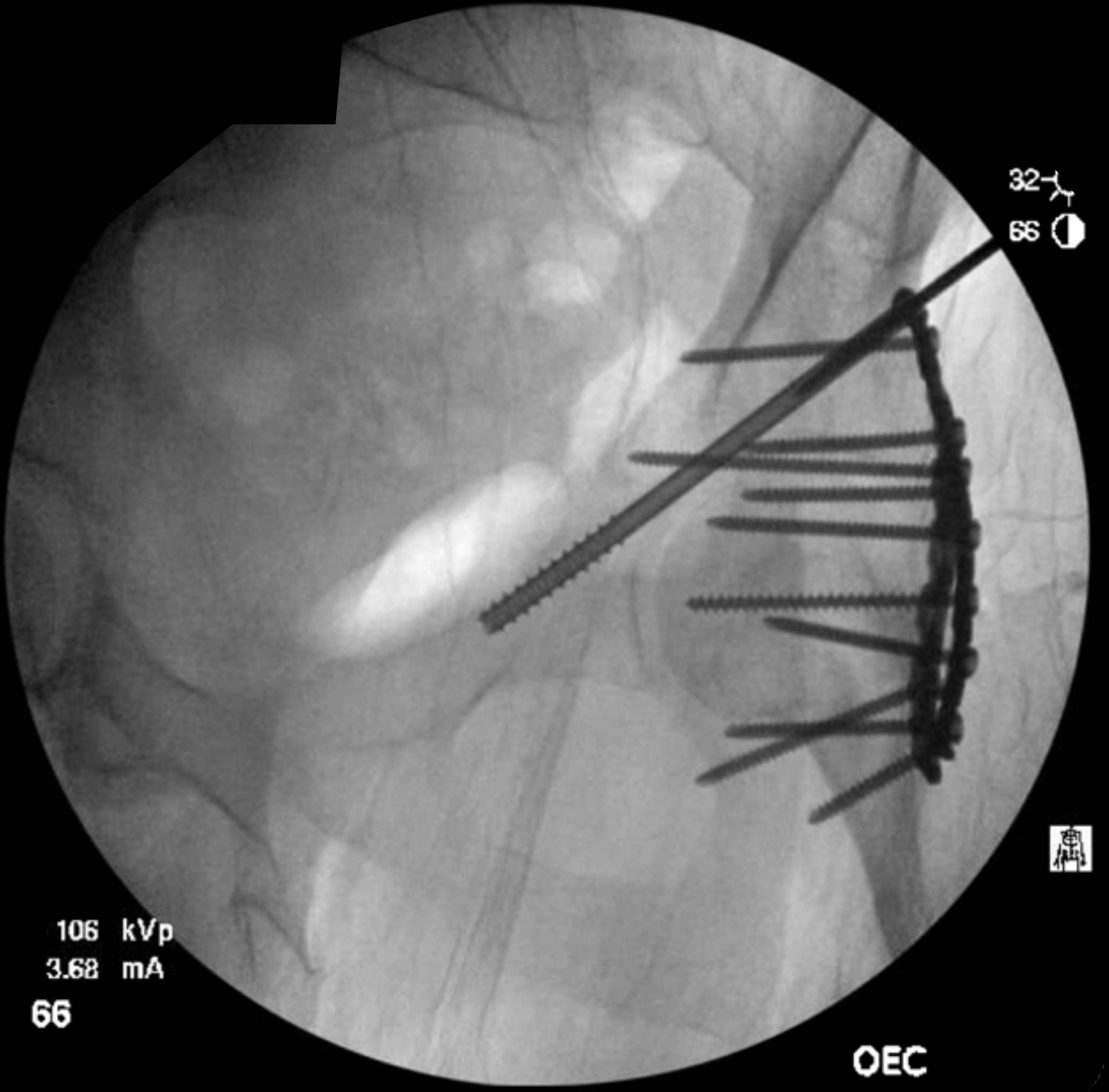

Insert the screw

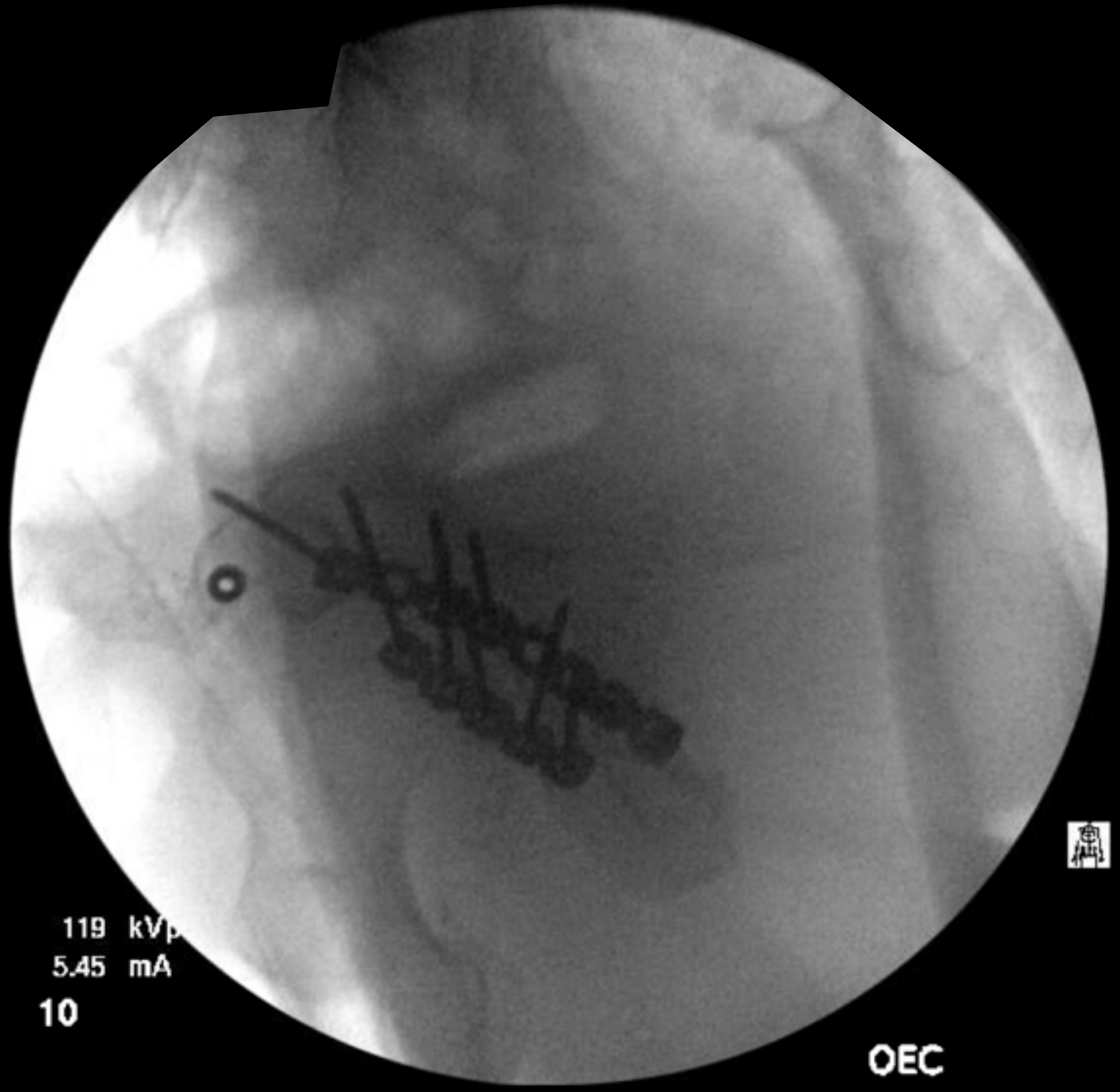

After screw insertion the anterior column view can be used to make sure the screw is not in the hip joint

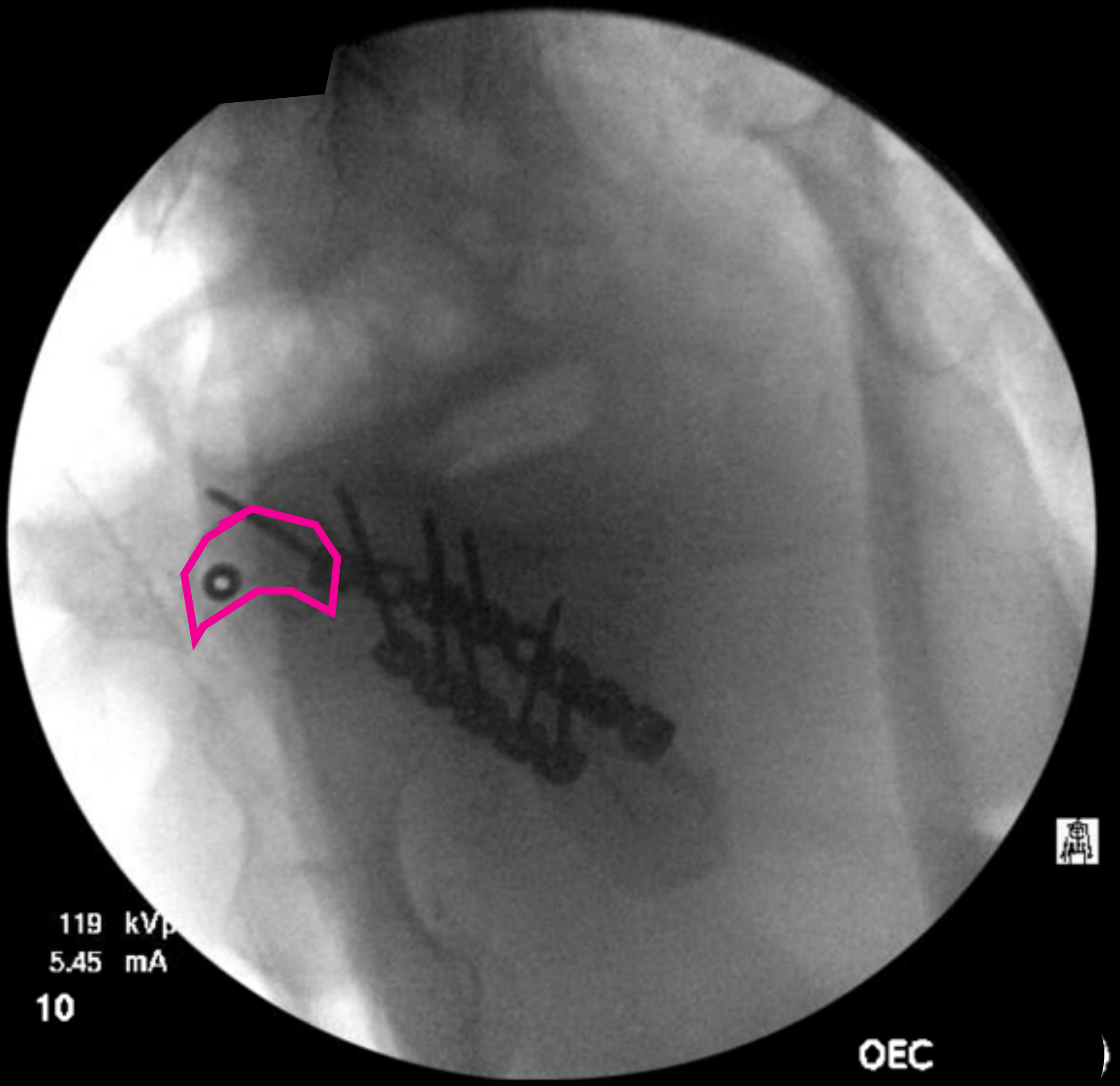

119 kVp
5.45 mA
10
OEC

CASE 1 62B1.2b + 61B2.3

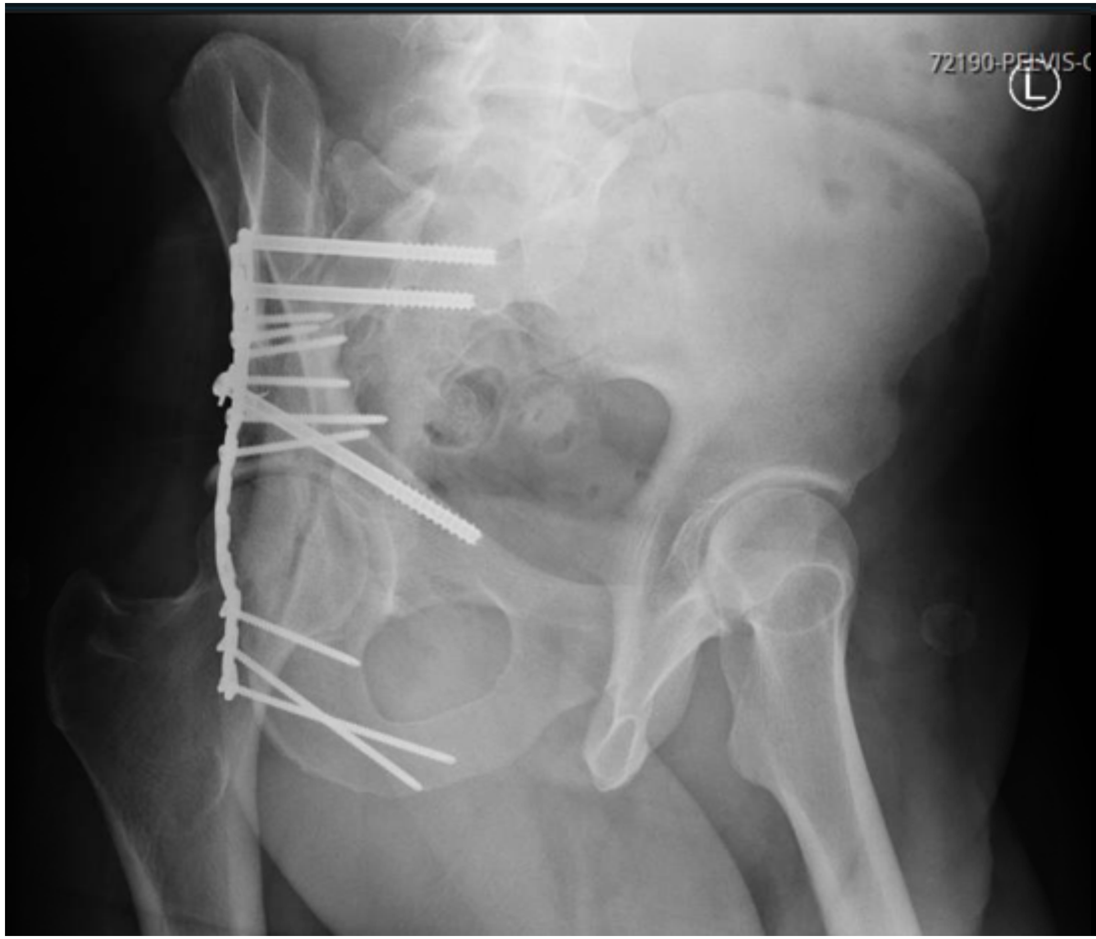

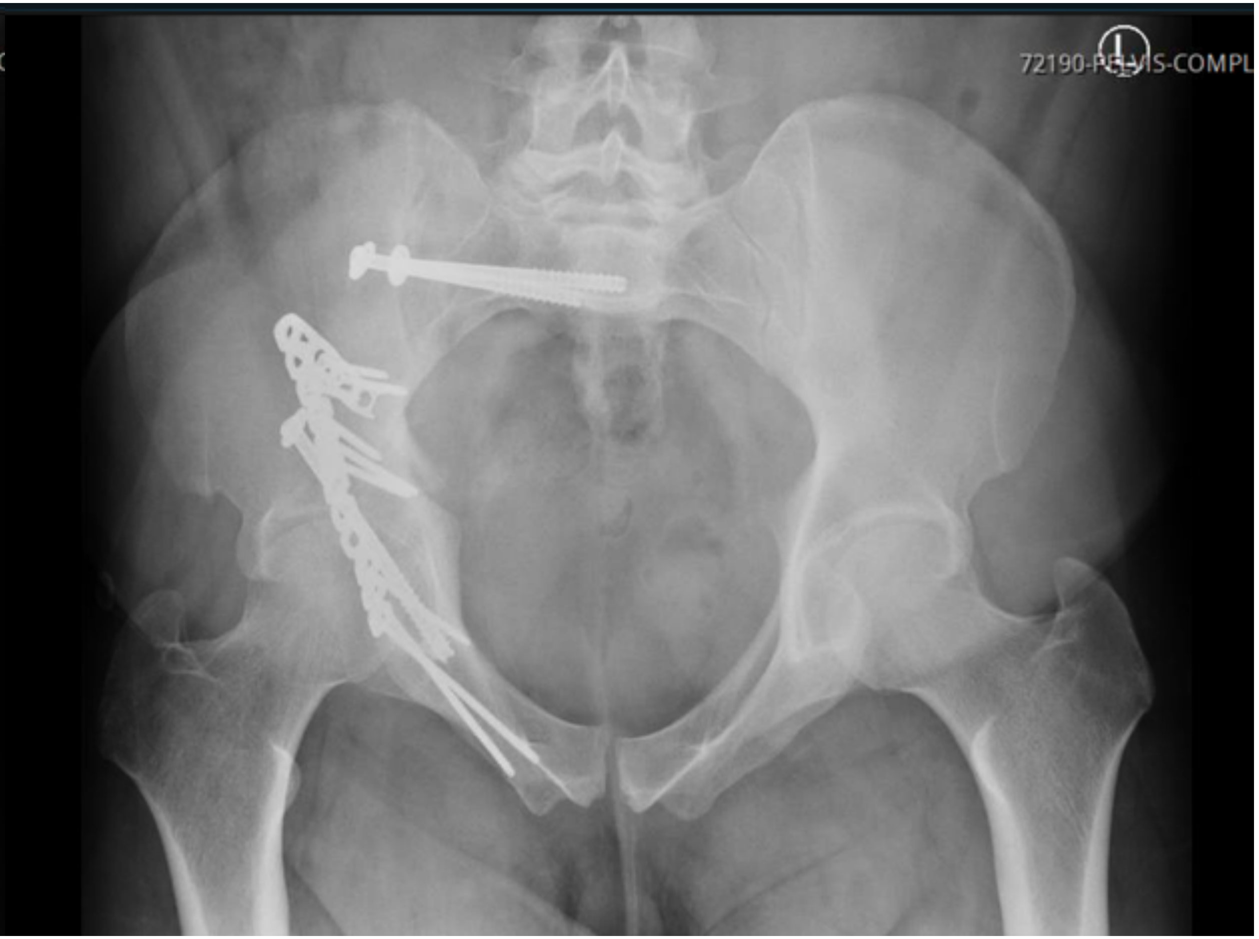

Case 2 62B1.2c + 61b3.3

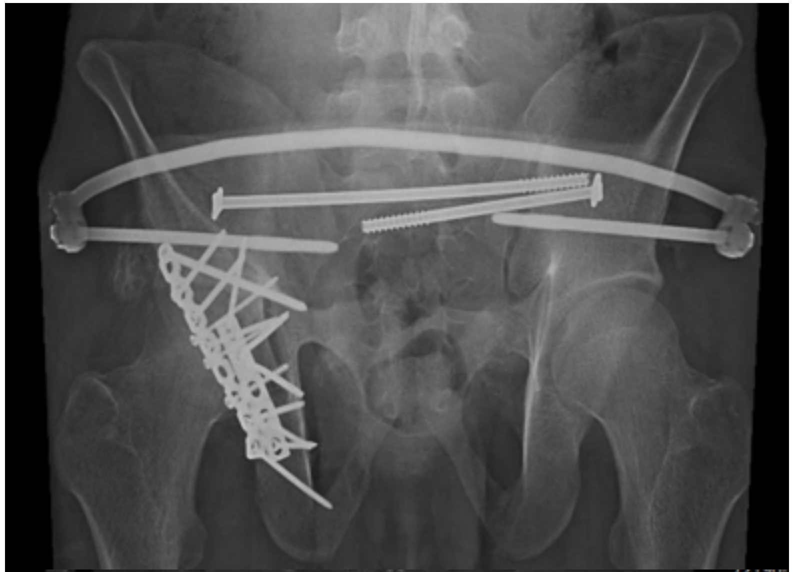

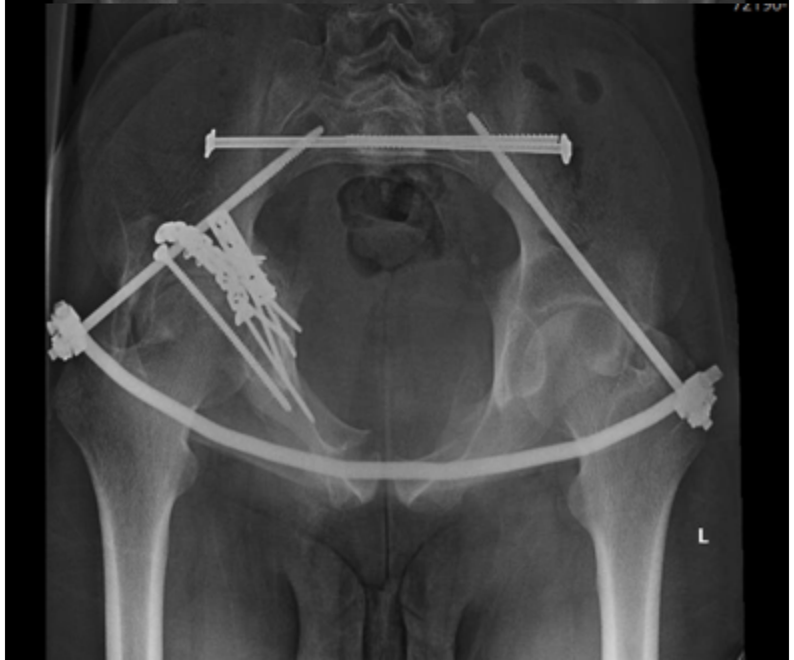

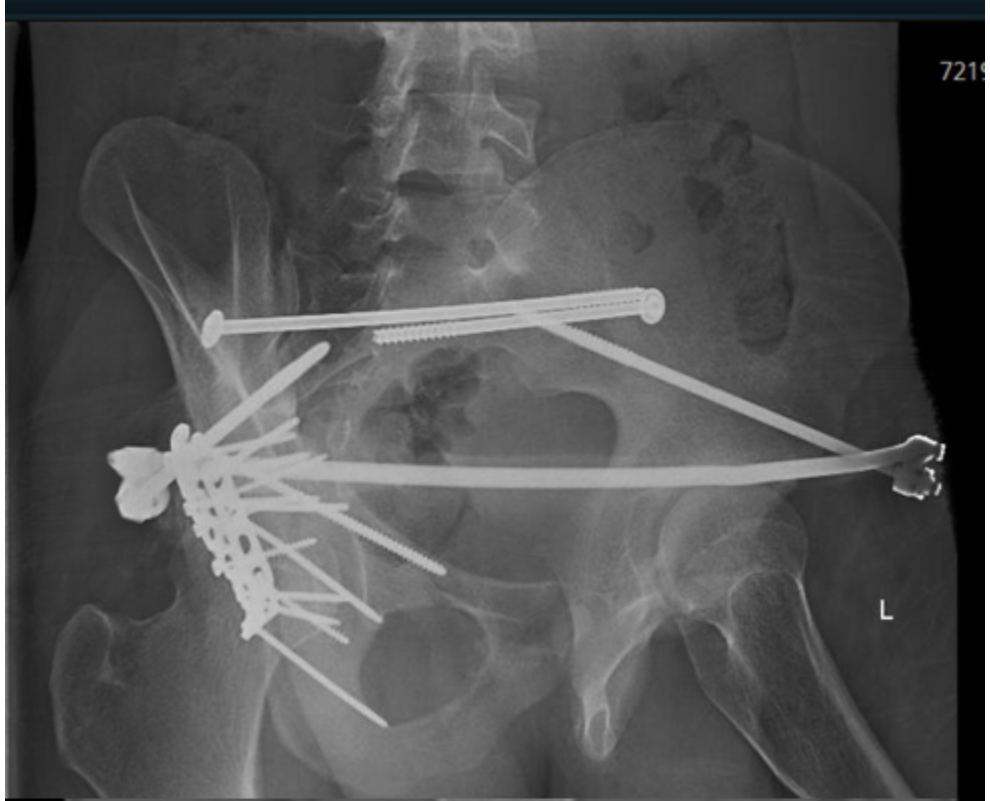

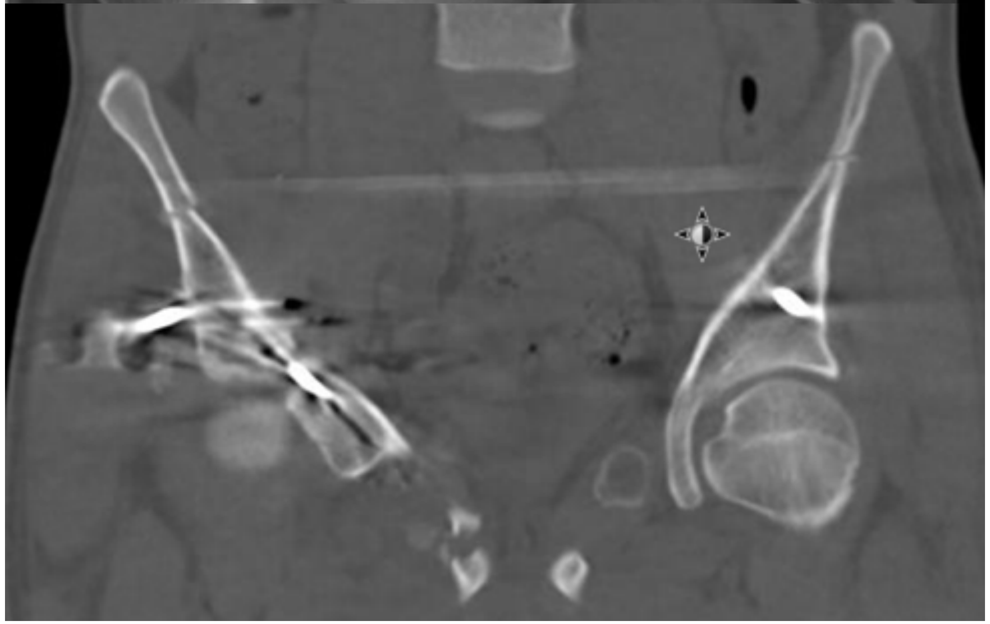

Case 3 62B1.2c

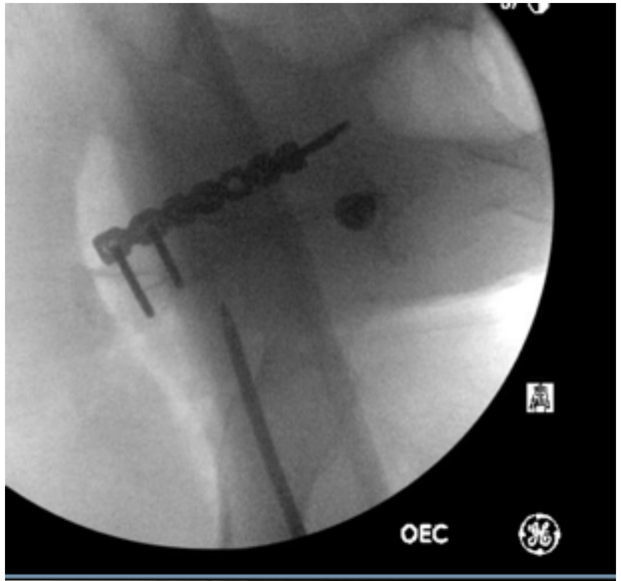

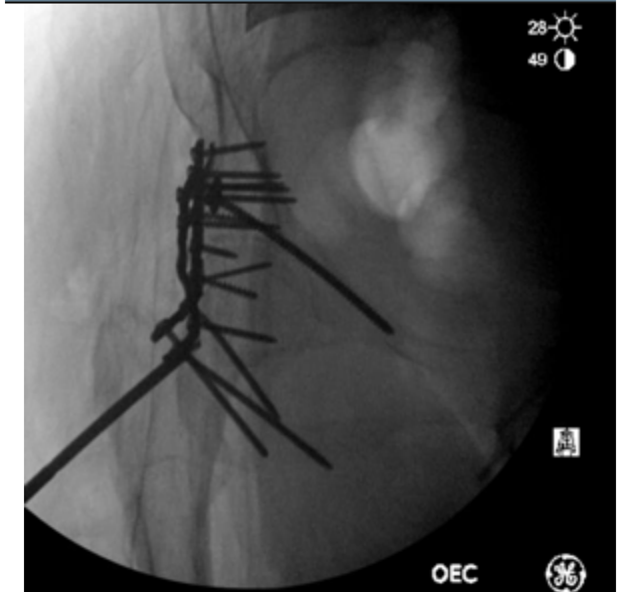

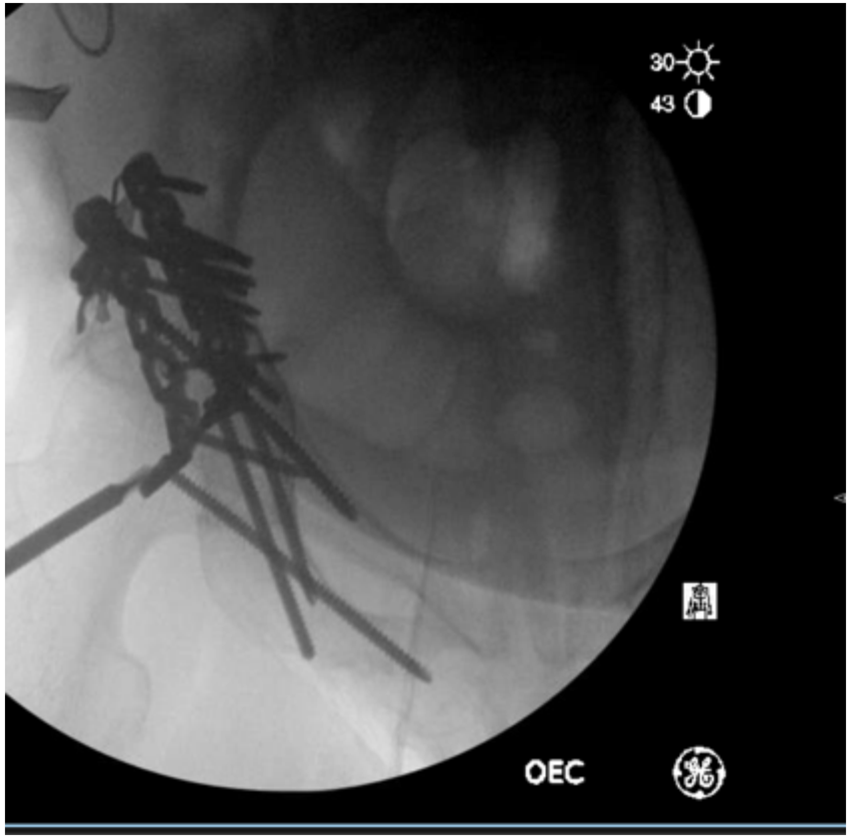

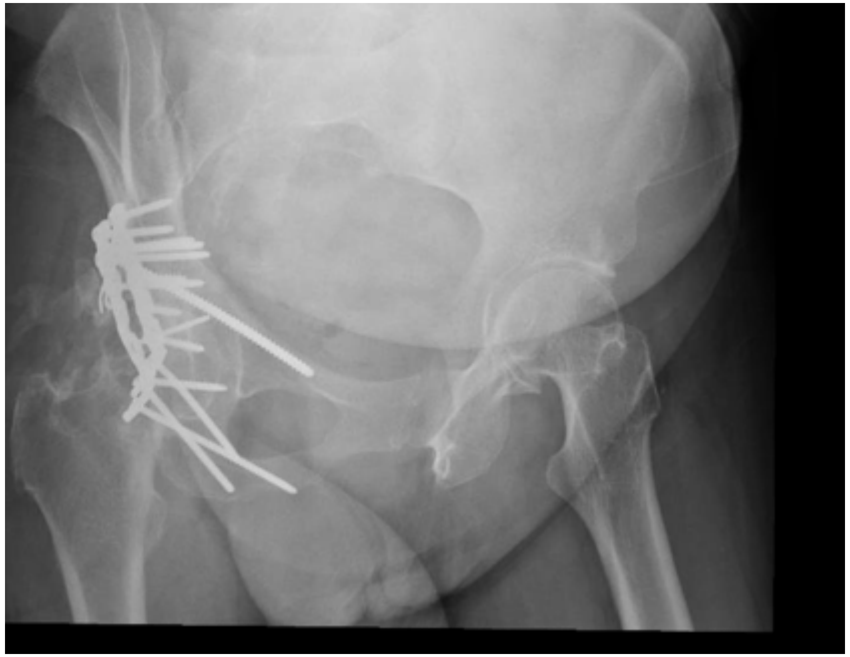

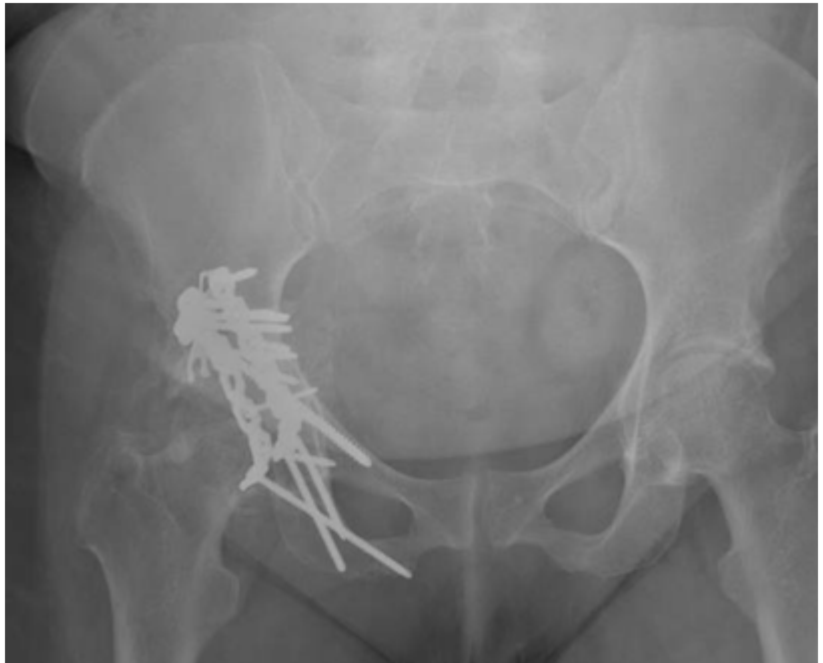

CASE 4 62B2.2c

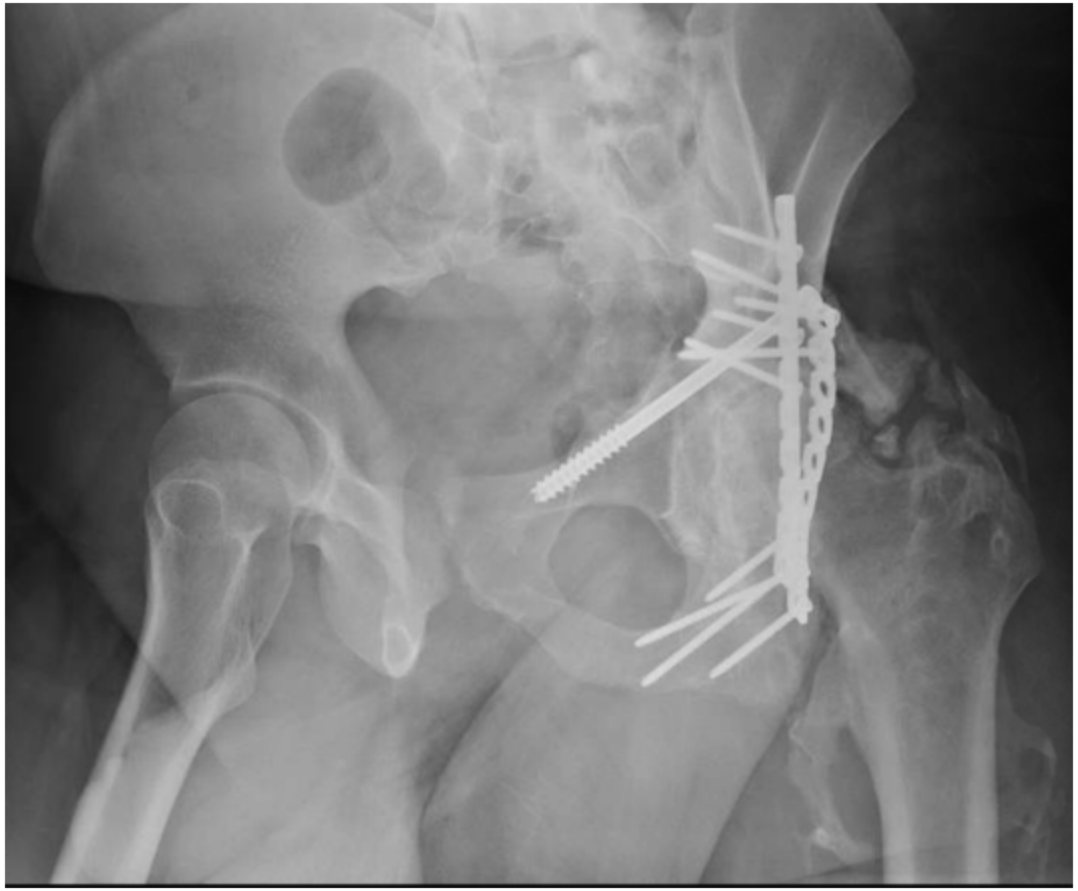

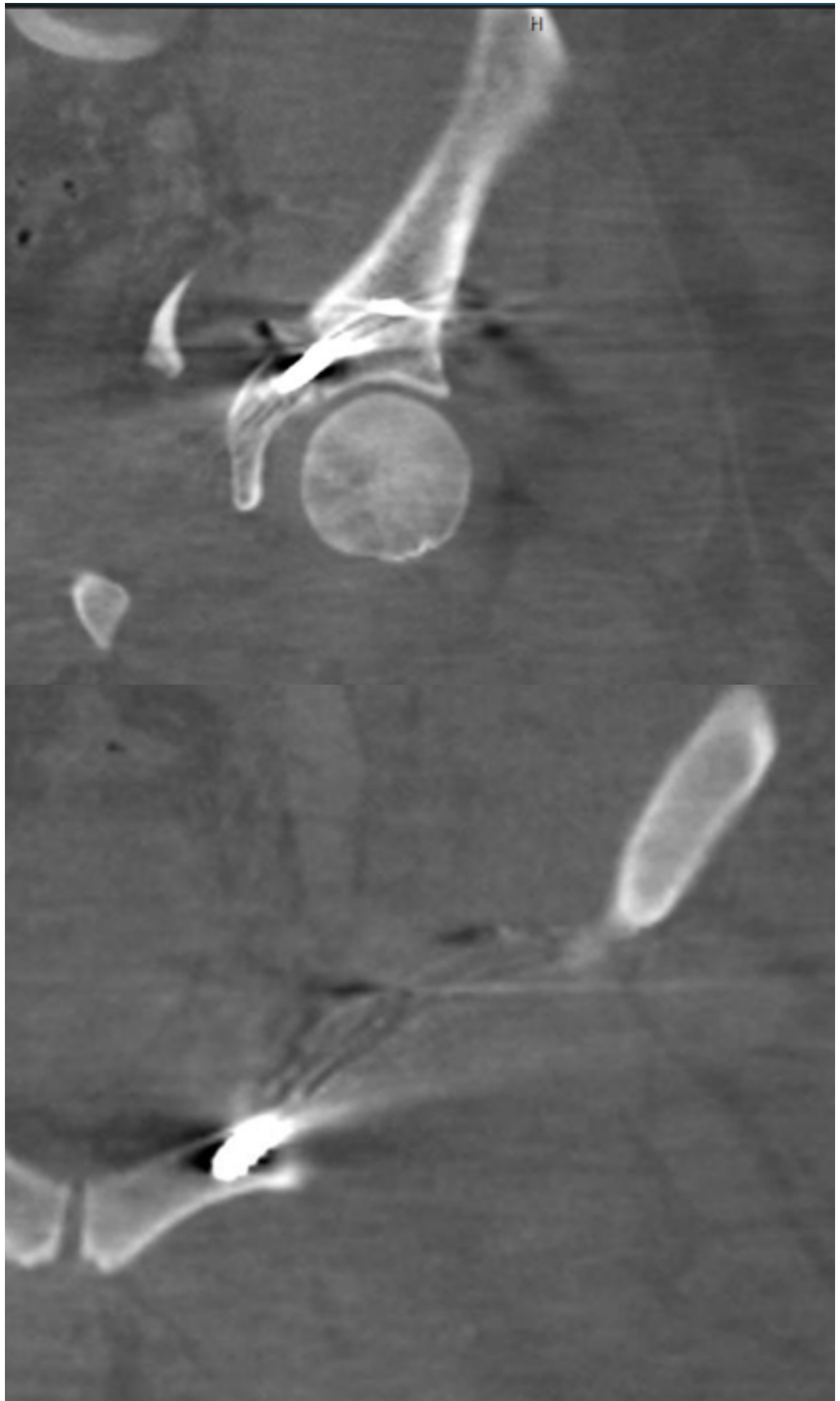

CASE 5 62B2.2 +61B3.1b

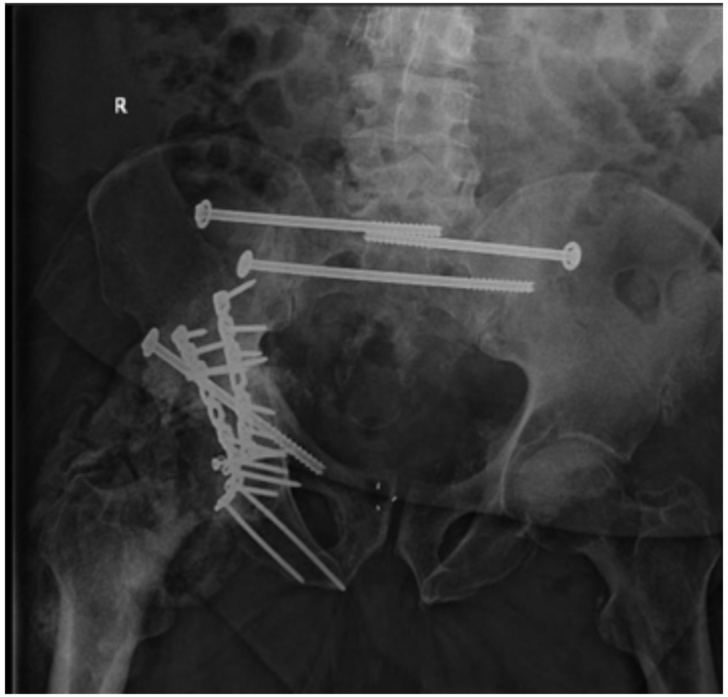

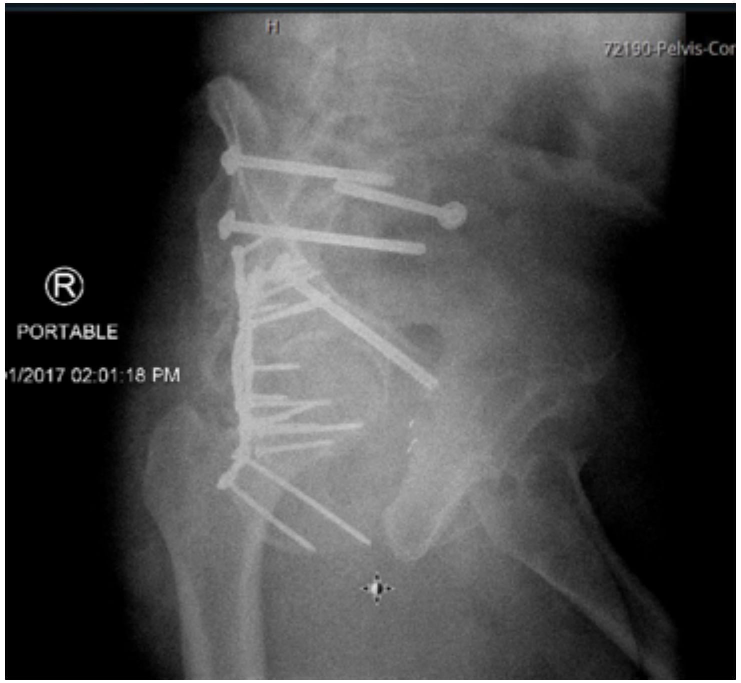

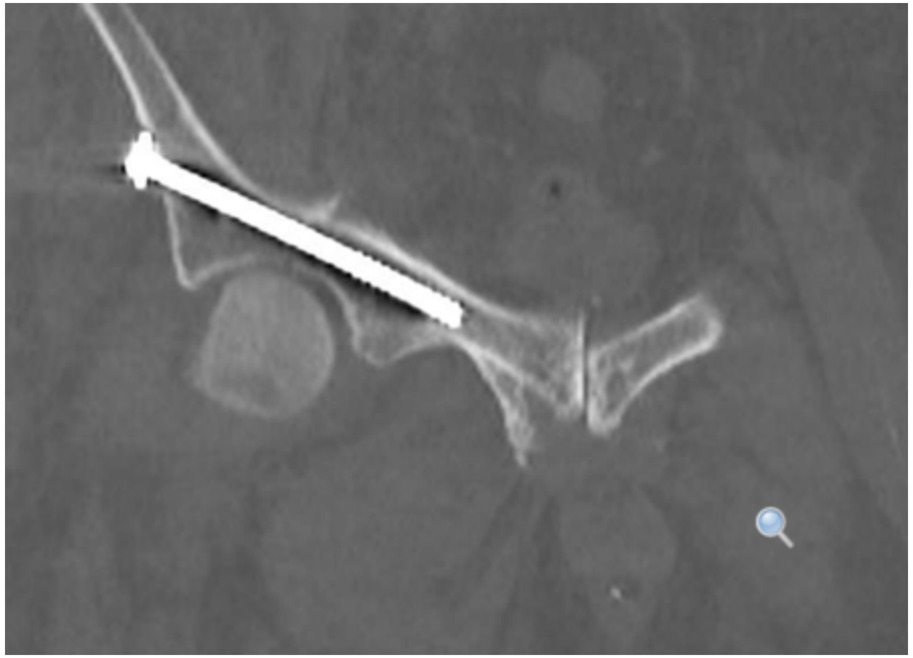

Case 6 62B2.2b+61C1.3

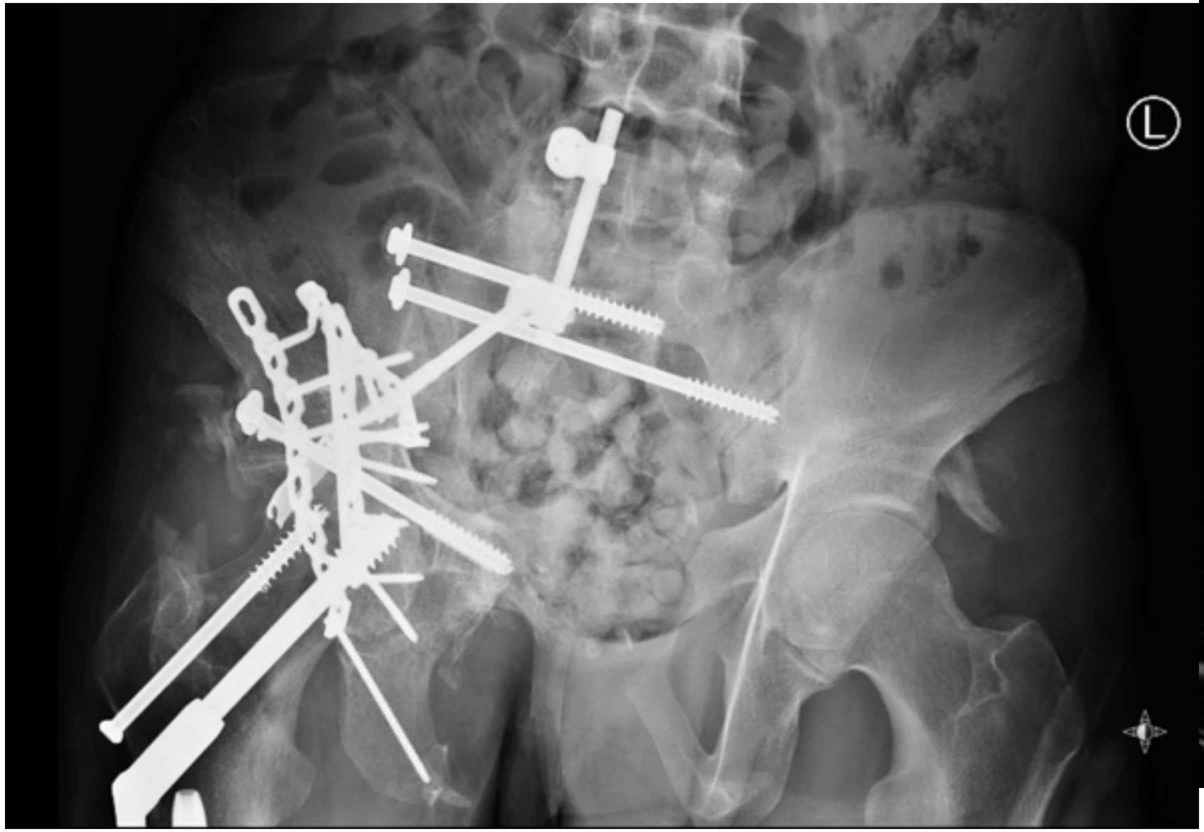

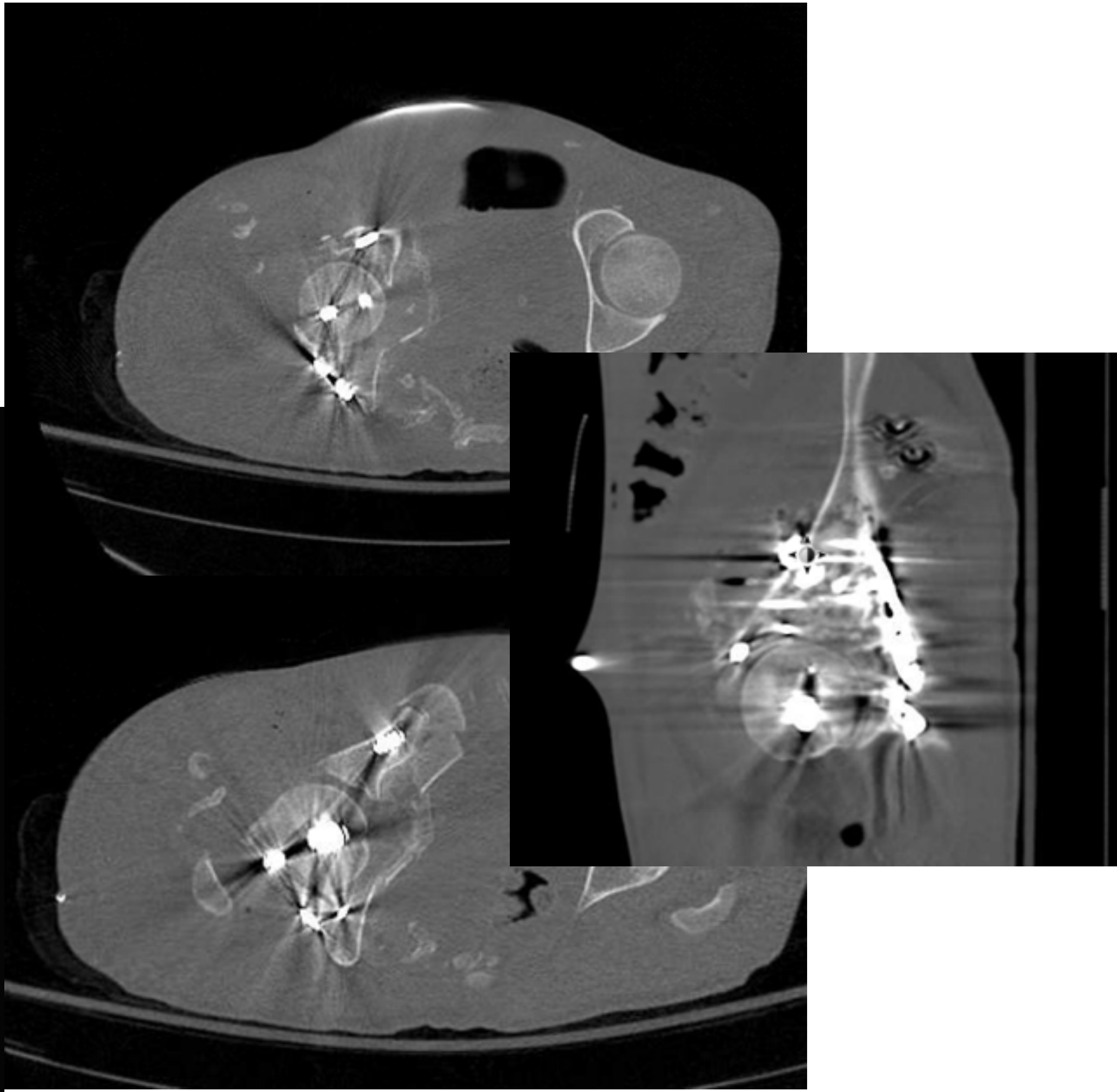

Case 7
61B2.3+62B1.2

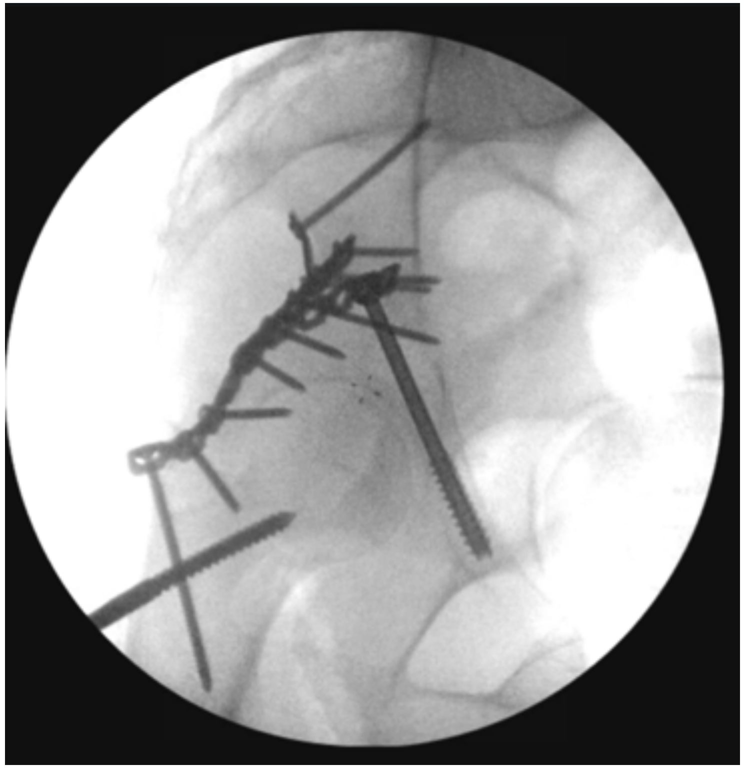

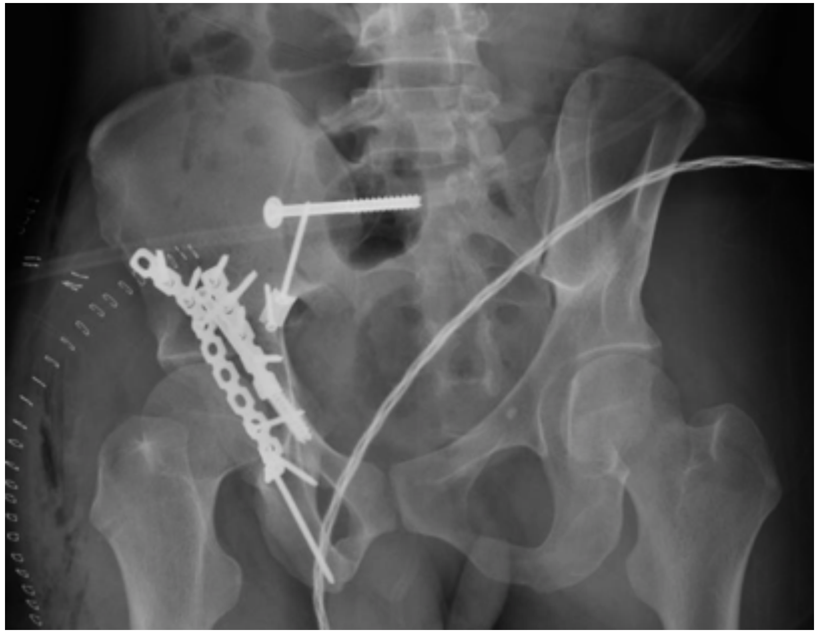

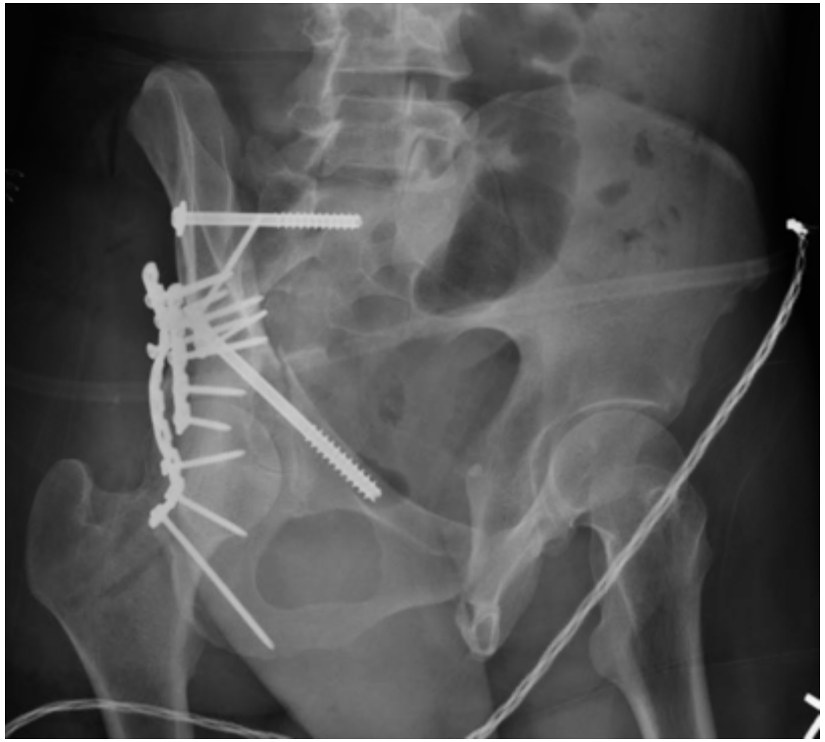

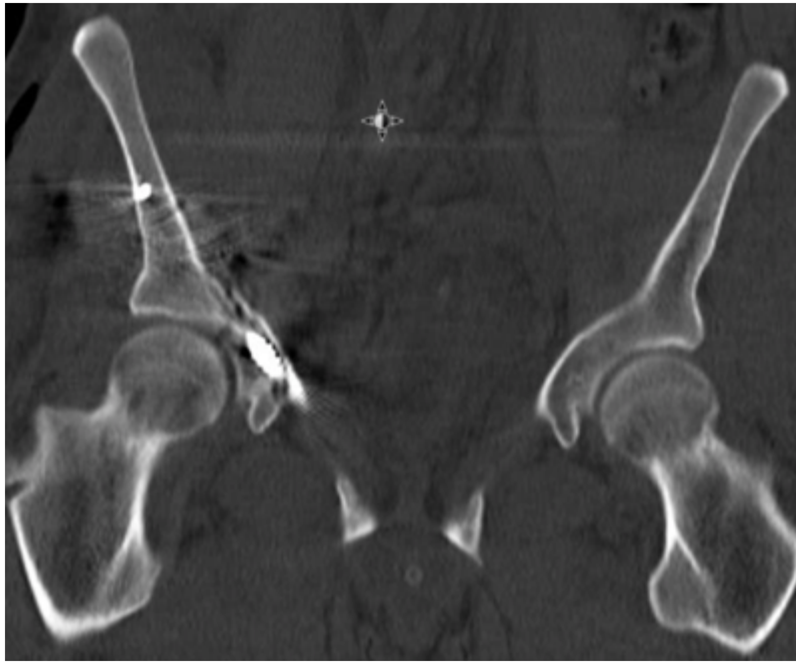

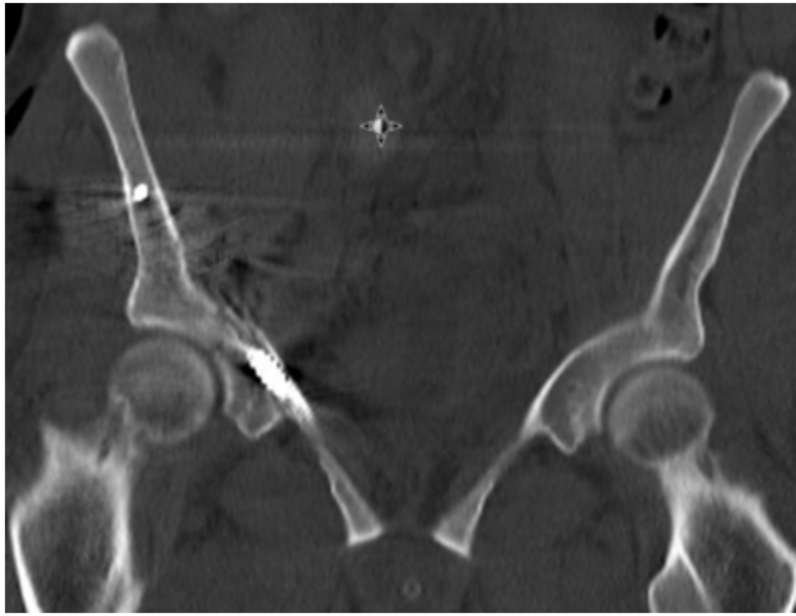

Case 11 62B1.2

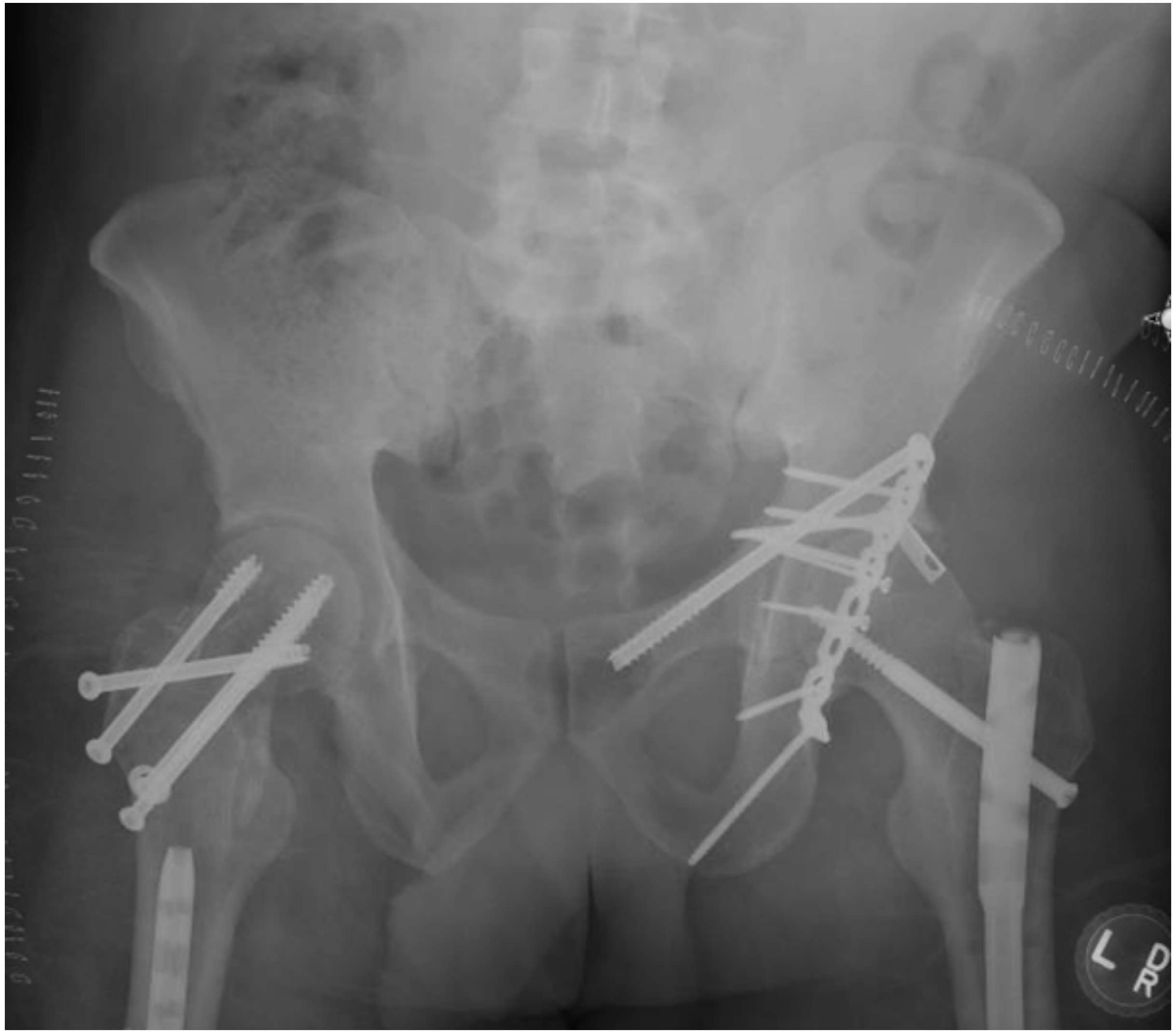

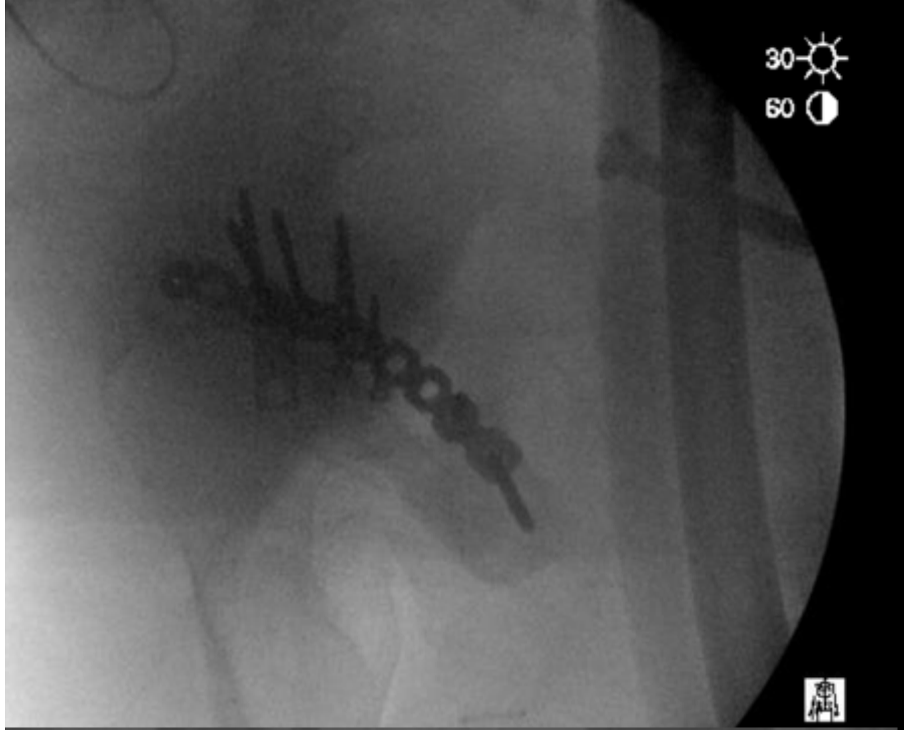

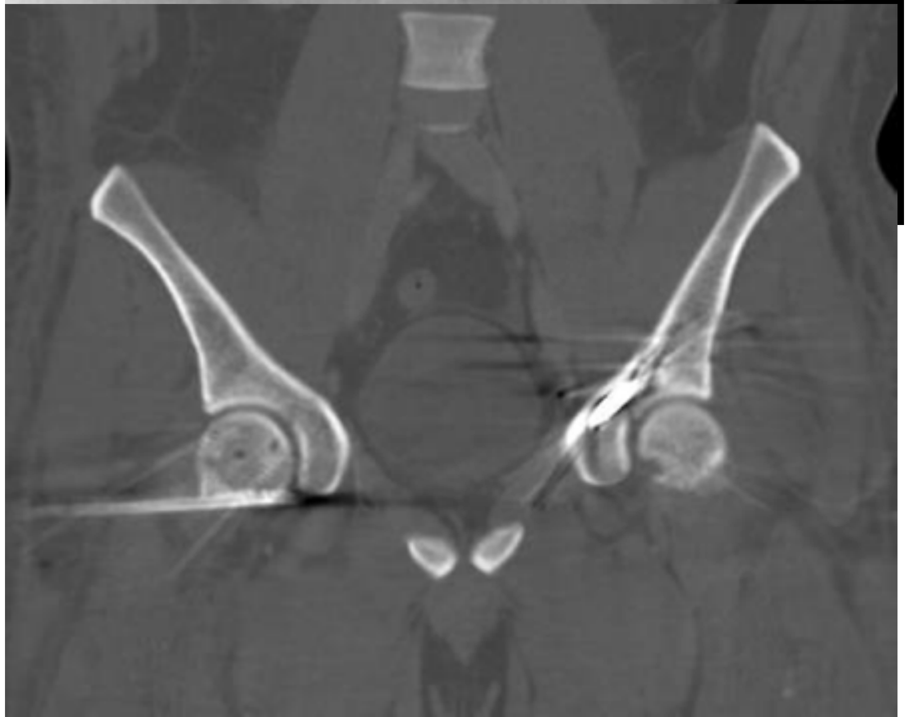

Case 12 62B1.2b

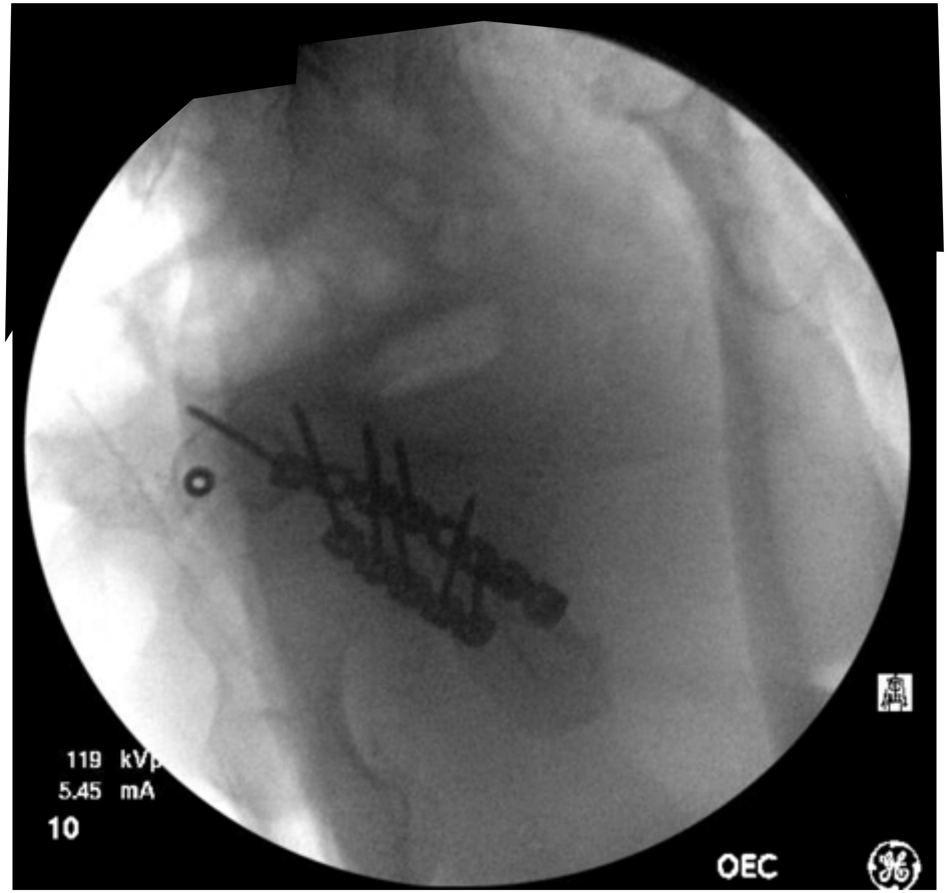

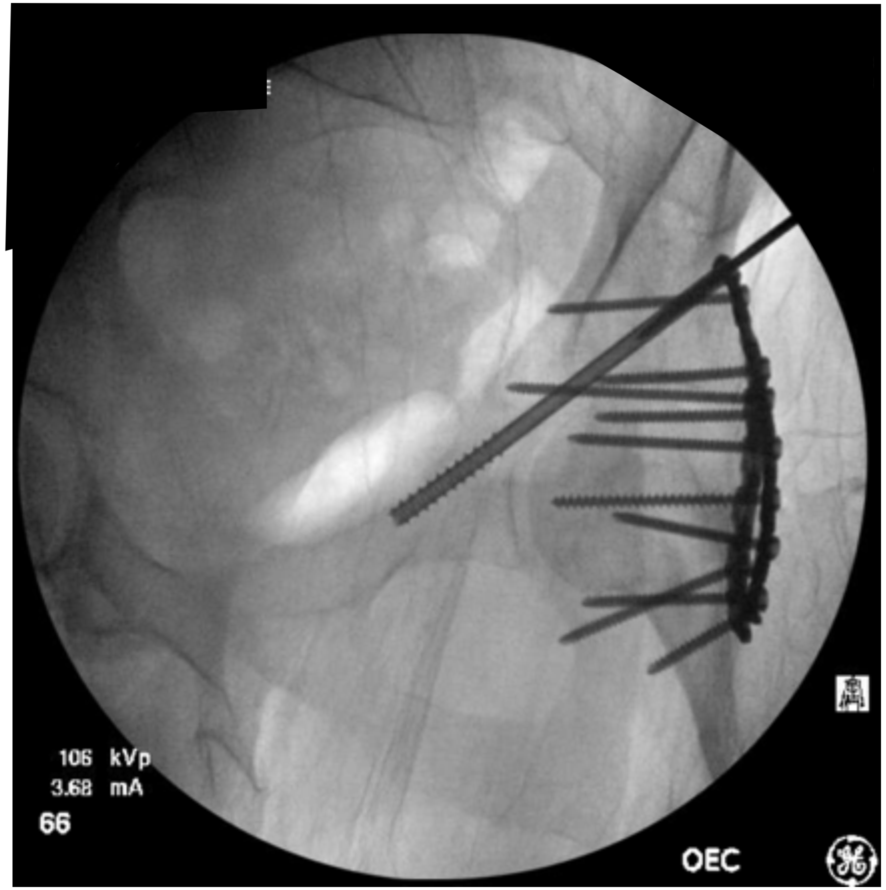

Case 14 62B1.2b

- Short screw but across the fracture

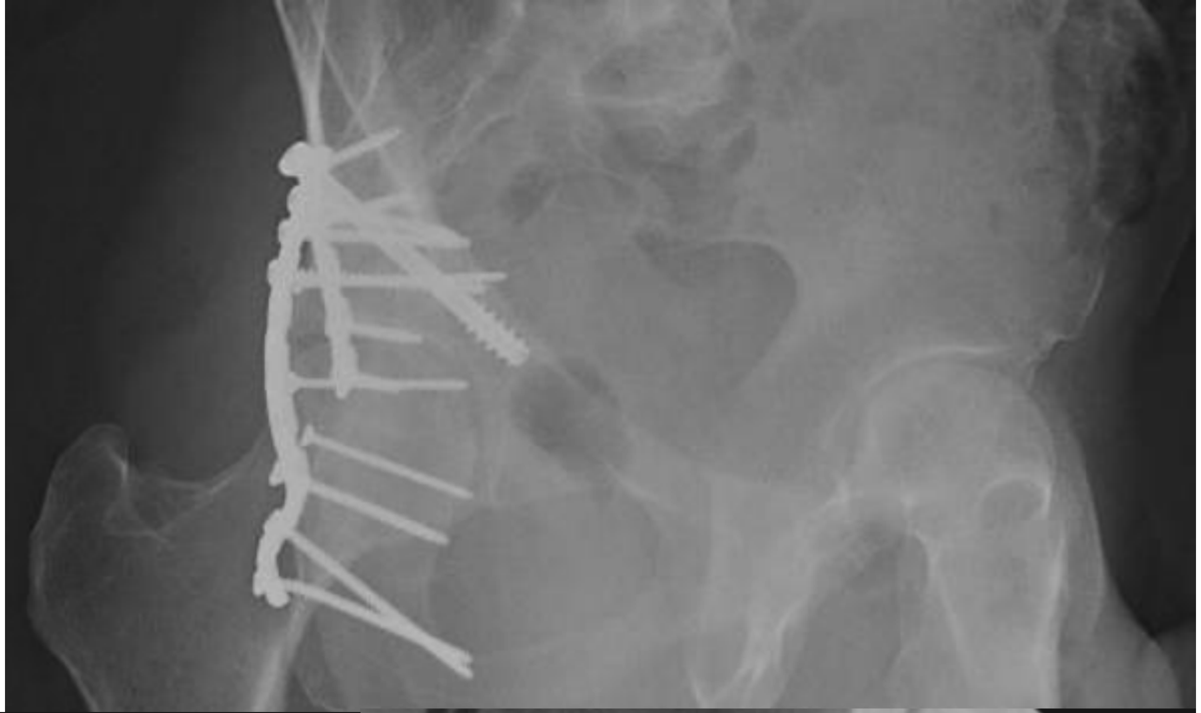

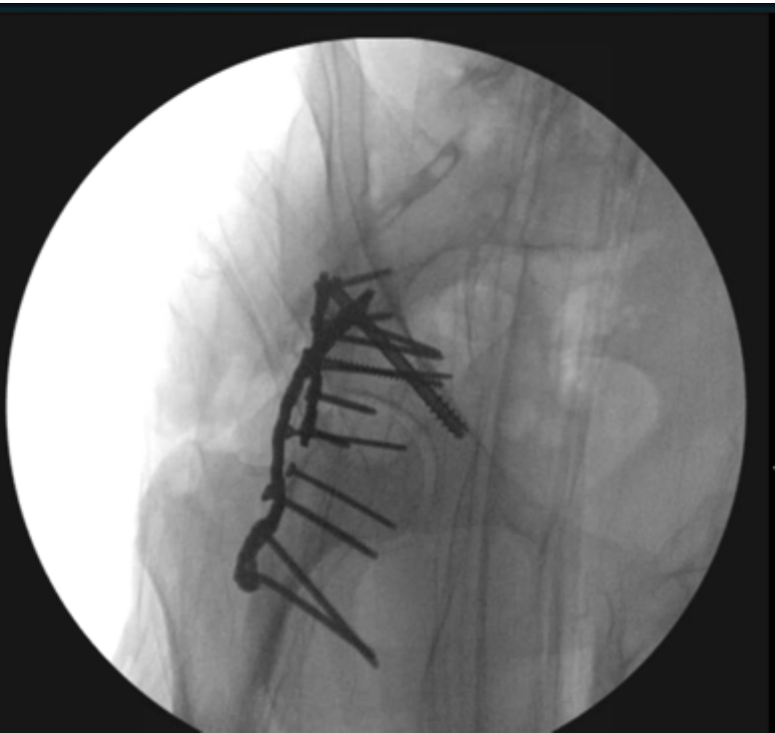

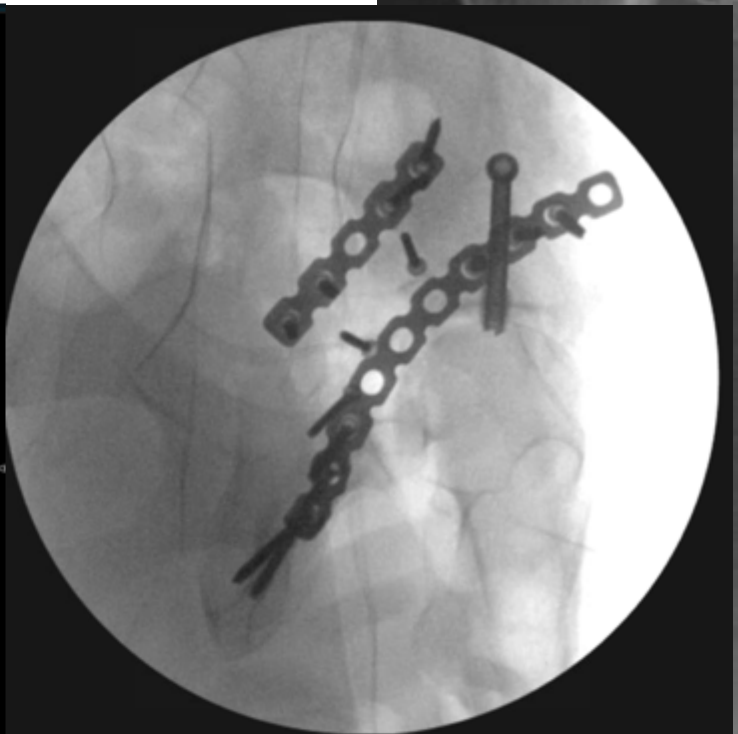

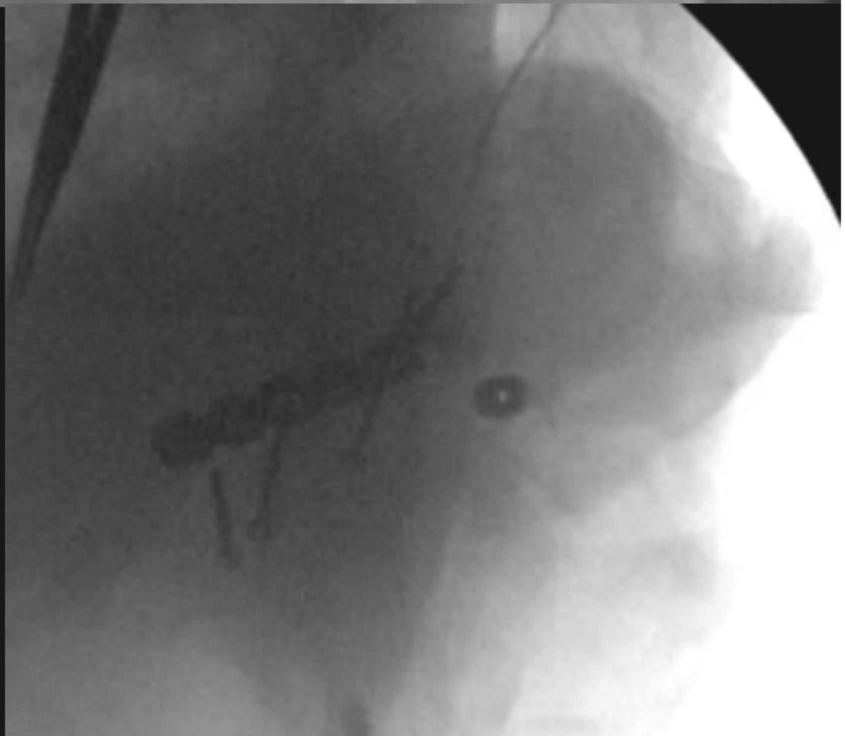

Case 15 rm 62B1.2b

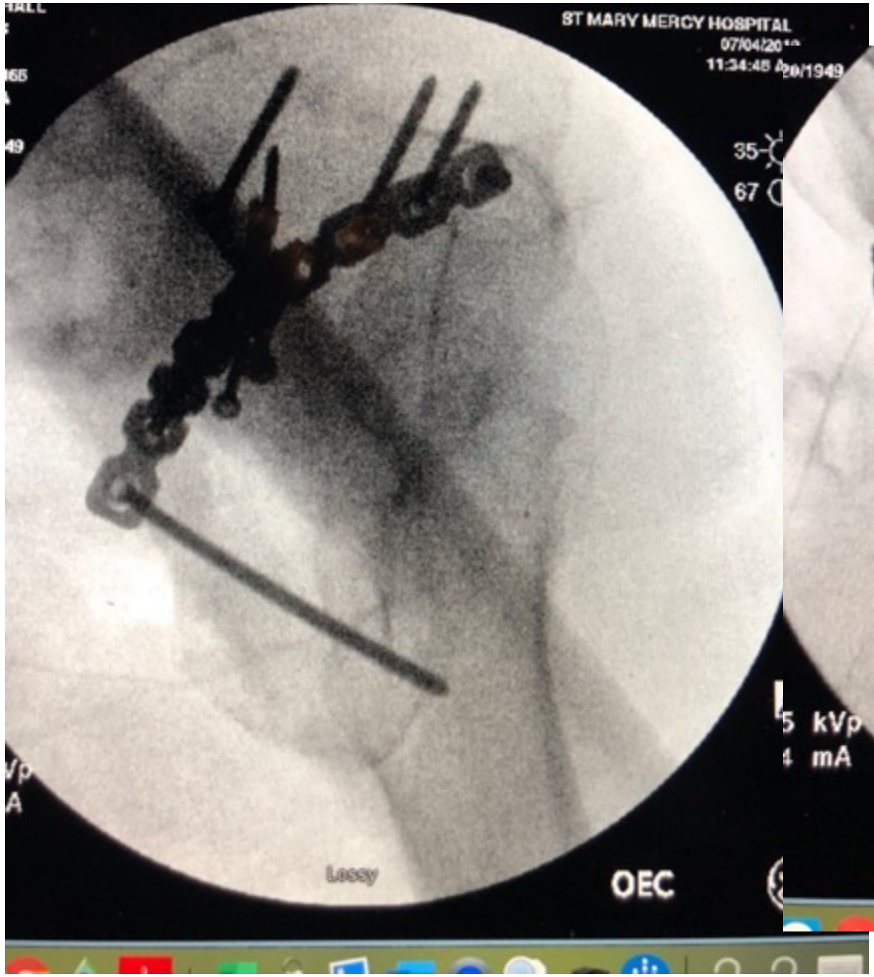

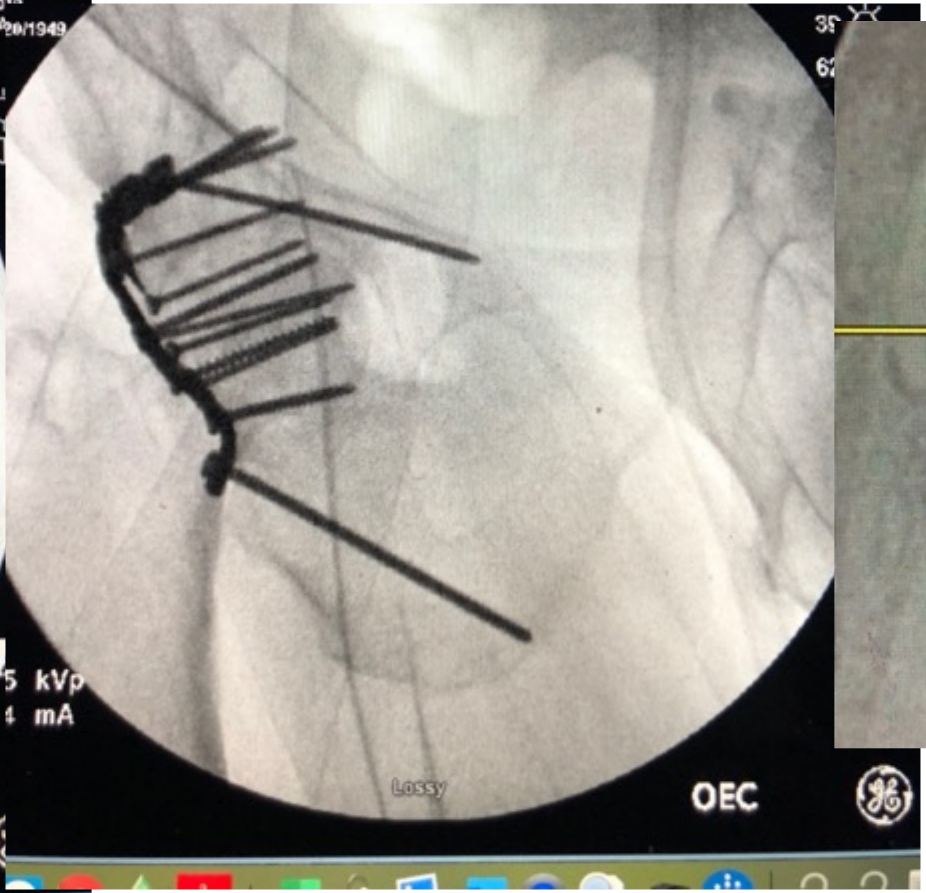

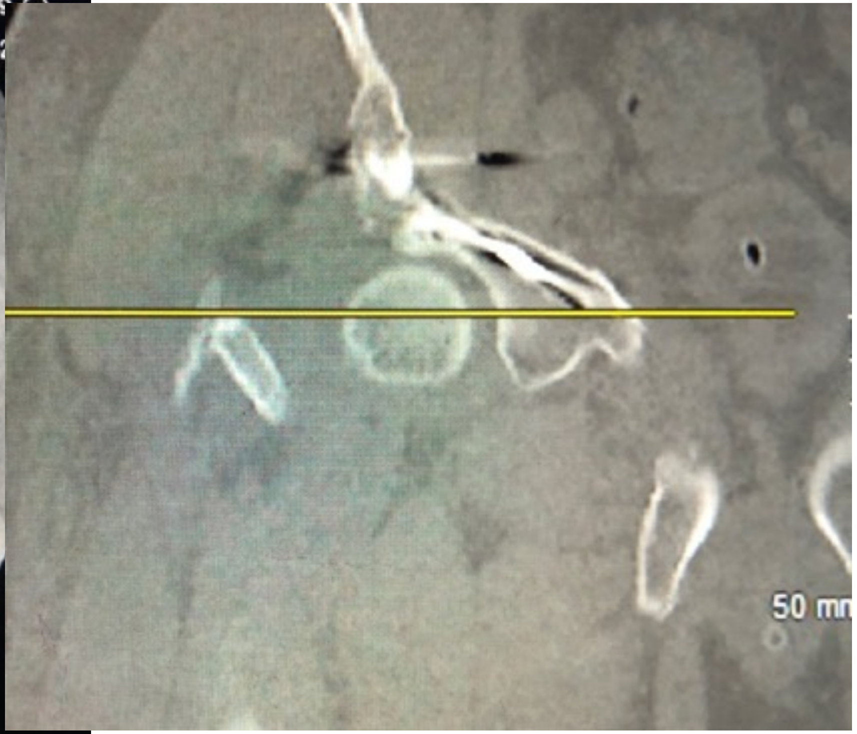

Case 16 61B2.1+62B1.2c

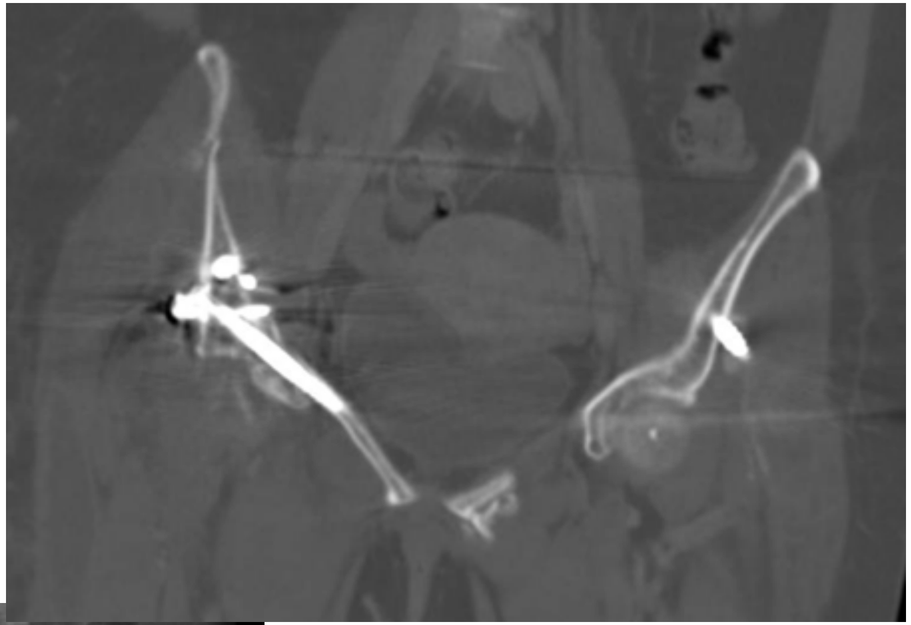

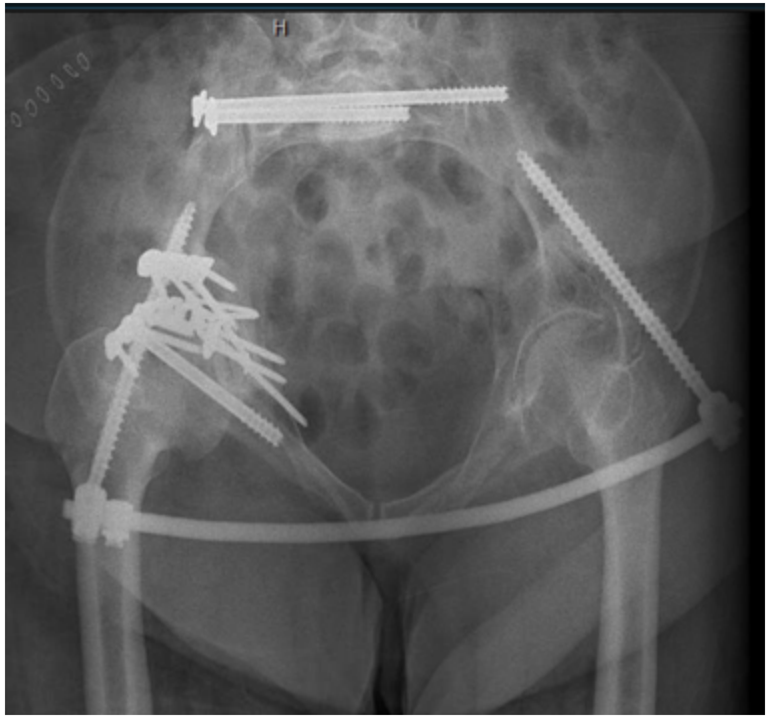

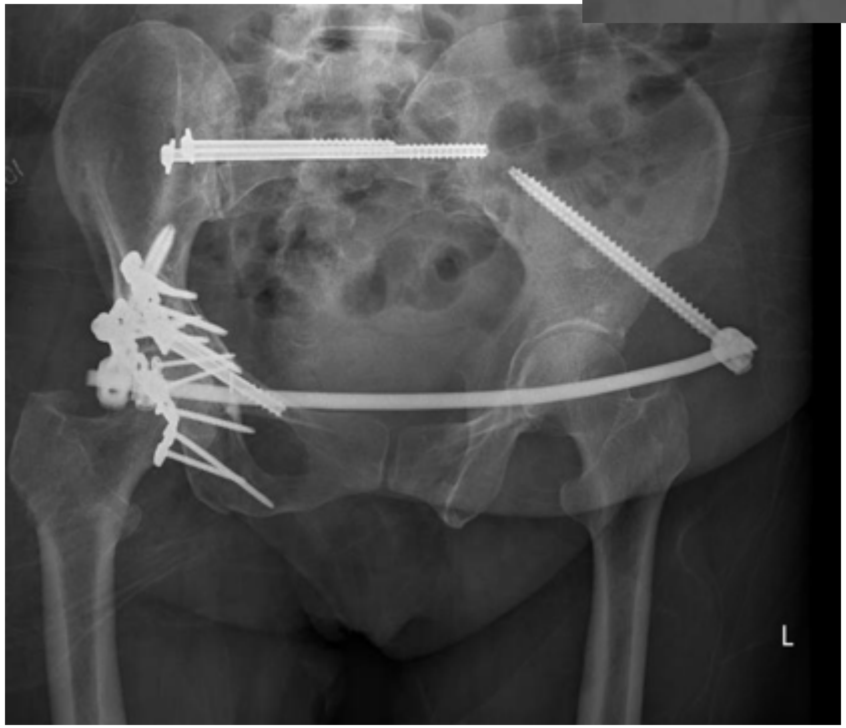

Supplement: Supplementary file 1 — Supplement 1. Supplemental digital content 1. [file sicotj-6-43-olm.pdf]
